# Defensin-like peptides in wheat analyzed by whole-transcriptome sequencing: a focus on structural diversity and role in induced resistance

**DOI:** 10.7717/peerj.6125

**Published:** 2019-01-08

**Authors:** Tatyana I. Odintsova, Marina P. Slezina, Ekaterina A. Istomina, Tatyana V. Korostyleva, Artem S. Kasianov, Alexey S. Kovtun, Vsevolod J. Makeev, Larisa A. Shcherbakova, Alexander M. Kudryavtsev

**Affiliations:** 1 Vavilov Institute of General Genetics, Russian Academy of Sciences, Moscow, Russia; 2 Moscow Institute of Physics and Technology, Dolgoprudny, Moscow Region, Russia; 3 All-Russian Research Institute of Phytopathology, B. Vyazyomy, Moscow Region, Russia

**Keywords:** Antimicrobial peptides, Defensins, Transcriptome, Induced resistance, Next-generation sequencing, Triticum kiharae, *Fusarium* spp., Elicitor

## Abstract

Antimicrobial peptides (AMPs) are the main components of the plant innate immune system. Defensins represent the most important AMP family involved in defense and non-defense functions. In this work, global RNA sequencing and *de novo* transcriptome assembly were performed to explore the diversity of defensin-like (DEFL) genes in the wheat *Triticum kiharae* and to study their role in induced resistance (IR) mediated by the elicitor metabolites of a non-pathogenic strain FS-94 of *Fusarium sambucinum*. Using a combination of two pipelines for DEFL mining in transcriptome data sets, as many as 143 DEFL genes were identified in *T. kiharae,* the vast majority of them represent novel genes. According to the number of cysteine residues and the cysteine motif, wheat DEFLs were classified into ten groups. Classical defensins with a characteristic 8-Cys motif assigned to group 1 DEFLs represent the most abundant group comprising 52 family members. DEFLs with a characteristic 4-Cys motif CX{3,5}CX{8,17}CX{4,6}C named group 4 DEFLs previously found only in legumes were discovered in wheat. Within DEFL groups, subgroups of similar sequences originated by duplication events were isolated. Variation among DEFLs within subgroups is due to amino acid substitutions and insertions/deletions of amino acid sequences. To identify IR-related DEFL genes, transcriptional changes in DEFL gene expression during elicitor-mediated IR were monitored. Transcriptional diversity of DEFL genes in wheat seedlings in response to the fungus *Fusarium oxysporum*, FS-94 elicitors, and the combination of both (elicitors + fungus) was demonstrated, with specific sets of up- and down-regulated DEFL genes. DEFL expression profiling allowed us to gain insight into the mode of action of the elicitors from *F. sambucinum.* We discovered that the elicitors up-regulated a set of 24 DEFL genes. After challenge inoculation with *F. oxysporum*, another set of 22 DEFLs showed enhanced expression in IR-displaying seedlings. These DEFLs, in concert with other defense molecules, are suggested to determine enhanced resistance of elicitor-pretreated wheat seedlings. In addition to providing a better understanding of the mode of action of the elicitors from FS-94 in controlling diseases, up-regulated IR-specific DEFL genes represent novel candidates for genetic transformation of plants and development of pathogen-resistant crops.

## Introduction

Like other multicellular organisms, plants have developed a sophisticated multi-layered defense system to combat invading pathogens and pests. Although plants are devoid of highly specific adaptive immune response characteristic of higher vertebrates, they share with animals the so-called innate immunity. It is based on both constitutive and inducible mechanisms preventing pathogen ingress and growth in host tissues. The crucial step in activation of defense responses in plants is perception of pathogens by two types of receptors – PRR (pattern recognition receptors) and the products of the resistance (R) genes (Jones & Dangl, 2006). Recognition of pathogens by both types of receptors activates signaling cascades that trigger defense gene expression. Defense reactions induced in plants include both physical and chemical protection, such as the reinforcement of cell walls through lignification and deposition of callose and synthesis of antimicrobial substances, such as phytoalexins and antimicrobial proteins and peptides (AMPs).

AMPs are small (<10 kDa) effector molecules of the plant immune system which provide a rapid and cost-effective “chemical” defense to circumvent infection (Broekaert et al., 1997; Odintsova & Egorov, 2012; Tam et al., 2015). Structurally diverse, they directly target a wide range of pathogens utilizing different modes of action and providing the first line of defense against invading microbes. Several families of plant AMPs have been discriminated. Defensins belong to the most ancient AMP family being discovered in fungi, plants, invertebrates and vertebrates (Lay & Anderson, 2005; Carvalho & Gomes, 2009). Plant defensins are small (40–50 amino acid residues), cysteine-rich, and cationic peptides. They display low sequence similarity except for conserved cysteine residues. The global fold of defensins comprises one α-helix and a triple-stranded antiparallel β-sheet stabilized by disulfide bonds. The plant defensins belong to the so-called cis-defensins characterized by two parallel disulphide bonds connecting the third β-strand to the α-helix (Shafee et al., 2016). Defensins are synthesized as precursor proteins. According to the precursor structure, two main classes of defensins have been discriminated (Lay & Anderson, 2005). The precursors of class 1 defensins consist of a signal peptide and a mature defensin domain. The precursors of class 2 defensins possess an additional C-terminal prodomain required for vacuolar targeting and detoxification of the peptide during its movement through the plant secretory pathway (Lay et al., 2014). Based on *in vitro* assays of isolated peptides, gene expression analysis and studies of transgenic plants, defensins were postulated to be involved in defense against fungal and bacterial pathogens and insect pests (Lay & Anderson, 2005; Carvalho & Gomes, 2009; Vriens, Cammue & Thevissen, 2014; Cools et al., 2017; Parisi et al., 2018), abiotic stress tolerance to salinity, drought, cold and metals (Lay & Anderson, 2005; Mirouze et al., 2006; Parisi et al., 2018), and in other, non-defense functions (Allen et al., 2008; Stotz, Spence & Wang, 2009; Shafee et al., 2016; Parisi et al., 2018).

Studies of innate immunity in plants showed that in addition to local responses, local infection increases resistance of the entire plant to a wide spectrum of pathogens, the phenomenon called systemic acquired resistance (SAR) (Ross, 1961; Ryals et al., 1996). Beneficial microbes, such as plant growth-promoting rhizobacteria (PGPR), mycorrhizal fungi (PGPF) and non-pathogenic strains of pathogenic fungi and their metabolites produce general or specific elicitors which are able to induce the same systemic resistance of the whole plant (ISR, induced systemic resistance) (Heil & Bostock, 2002; Gozzo, 2003; Choudhary, Prakash & Johri, 2007). The onset of SAR is accompanied by a local and systemic increase in the levels of salicylic acid (SA) (Métraux et al., 1990) and up-regulation of genes encoding pathogenesis-related (PR) proteins (Maleck et al., 2000). Some of them display antimicrobial activity and are supposed to contribute to enhanced resistance. ISR depends on the plant hormones jasmonic acid (JA) and ethylene (ET) and is not associated with PR-protein accumulation (Hoffland et al., 1995; Pieterse et al., 1996). However, some PGRP and PGPF induce SA-dependent or both SA- and JA/ET- dependent systemic resistance (Sultana et al., 2009; Molitor et al., 2011; Gkizi et al., 2016). Despite considerable advances in understanding of the molecular mechanisms of interactions between plants and pathogenic and nonpathogenic microorganisms in induced resistance, the details of the processes involved, especially the role of antimicrobial peptides, remain largely unknown. Studies of induced resistance are of prime importance for elucidation of the molecular mechanisms of plant immunity. In addition, they have significant practical applications for the development of novel disease control measures based on activation of plant’s own defense mechanisms by microbial biocontrol agents. Although the economic potential of biocontrol strains in increasing disease resistance in crops is enormous, the molecular components and mechanisms of resistance induced by biocontrol strains and their metabolites remain largely enigmatic that hampers their practical application.

Earlier, we showed that the intracellular elicitor metabolites produced by the biocontrol *Fusarium sambucinum* (isolate FS-94) protect wheat from *Stagonospora nodorum* causing glume/leaf blotch as well as from multiple fungi belonging to the pathogenic root rot complex (*F. culmorum*, *F. avenaceum*, *F. oxysporum*, *F. sporotrichioides*, *F. gibbosum*, and *Bipolaris sorokiniana*) (Shcherbakova et al., 2012; Shcherbakova et al., 2018) and induce systemic resistance in plants (Shcherbakova et al., 2011; Shcherbakova et al., 2018). In this work, to further analyze the mode of action of the resistance elicitors from *F. sambucinum* and to elucidate the role of defensins, one of the most important AMP family, in induced resistance (IR), we used global transcriptome sequencing (RNA-seq) of wheat seedlings treated with the elicitors and displaying IR. Using a combination of two pipelines developed for defensin-like (DEFL) genes identification in transcriptome data sets, we explored the repertoire of DEFL genes in the wheat *Triticum kiharae* and monitored changes in DEFL gene expression in infected, elicitor-treated and IR-expressing seedlings. As a result, we revealed dozens of novel DEFL genes in wheat and determined defensin sets associated with mode of action of *F. sambucinum* elicitor metabolites and IR for future functional studies and practical applications.

## Materials & Methods

### Biological material

Seeds of the wheat *Triticum kiharae* Dorof. et Migush., which is a synthetic allopolyploid produced by crossing *Triticum timopheevii* (AAGG) with *Aegilops tauschii* (DD), were obtained from the collection of the Vavilov Institute of General Genetics, Russian Academy of Sciences (Moscow, Russia). The fungus *F. oxysporum* strain 137 was from the collection of the All-Russian Research Institute of Phytopathology (Moscow region, Russia). Elicitor metabolites from *F. sambucinum* (strain FS-94) were isolated as described for the resistance-inducing fraction of *F. oxysporum* strain CS-20 (Shcherbakova et al., 2016).

### Experimental design

Wheat seeds (250) were immersed in 0.5% KMnO_4_ for 10 min, washed thoroughly with sterile distilled water (sdW) and incubated at 20–22 °C for 16 h, whereupon imbibed seeds were divided into two portions (100 seeds in each), placed on sterile paper filters in Petri dishes (25 seeds per dish) and treated with sterilized *F. sambucinum* metabolites (50 µl per seed) or sdW for 2.5–3 h under aseptic conditions. One half of elicitor- and sdW-treated seeds were inoculated with *F. oxysporum* strain 137 spore suspension (10^6^ spores/ml, 100 µl per kernel). Non-inoculated sdW-treated seeds were used as control. After the treatments, 200 germinated seeds were grown for 3 days at 20–22 °C (the first day in the dark, and then two days under long-day conditions (16 h day/8 h night)), harvested, immediately frozen in liquid nitrogen and stored at −80 °C until total RNA isolation.

Thus, four samples of young seedlings were obtained: (1) control group: seeds were treated with sterile water; (2) induced sample: seeds were treated with elicitor metabolites of *F. sambucinum* isolate FS-94; (3) infected sample: seeds were treated with sterile water and further infected with *F. oxysporum;* (4) IR-displaying sample: seeds were treated with FS-94 metabolites and further infected with the pathogenic strain 137 of *F. oxysporum*.

### Plant protection assay

The protective effect of the resistance-inducing metabolites from *F. sambucinum* (isolate FS-94) was assayed as described (Shcherbakova et al., 2018). Briefly, 50 surface-sterilized *T. kiharae* seeds were incubated in the FS-94 elicitor metabolites for 3 h as described above and infected with *F. oxysporum* by soaking in the fungal spore suspension for 30 min, whereupon, seeds were placed on filter paper towels, which were rolled up and put in beakers with distilled water. Wheat seedlings were grown in a climate chamber at 22 °C (16-h day) and 16 °C (8-h night). After 12 days, the number of infected seedlings was counted, and disease symptom severity was evaluated. Seeds treated with distilled water served as control.

### RNA isolation

Total RNA was isolated from 150 µg of plant material using the Plant RNA Isolation Aid kit (Ambion, ThermoFisher) according to the manufacturer’s protocol. The quality of total RNA samples was checked with NanoDrop 2000 (Thermo Fisher, Waltham, MA, USA) and Agilent 2100 Bioanalyzer (Agilent, Santa Clara, CA, USA). One half of each total RNA sample was used for generation of four cDNA libraries for Illumina HiSeq2500 sequencing, the remaining half, for RT-PCR validation.

### Library construction and NGS

The mRNA was isolated from the total RNA of four *T. kiharae* samples using RNA purification beads followed by fragmentation and priming for cDNA synthesis as recommended by the manufacturer (Illumina, San Diego, CA, USA). For double-stranded cDNA synthesis, the SuperScript Double-Stranded cDNA Synthesis kit (Invitrogen, USA) was used, further purification was accomplished using Agencourt AMPure XP beads (Beckman Coulter, Inc, Brea, CA, USA). End repairing and 3′-end adenylation were performed following the RNA adapter ligation. Upon enrichment of DNA fragments library templates were validated using Agilent 2100 Bioanalyzer (Agilent). Clonal clusters were created from DNA library templates using TruSeq PE Cluster Kit v2 and cBot automated system (Illumina). Clusters obtained were used to carry out paired-end runs by Genome Analyzer *IIx* (Illumina). Illumina HiSeq2500 sequencing was carried out on the equipment of EIMB RAS “Genome” Center.

### Analysis of transcriptome sequencing data

Read trimming and removal of adapter sequences was performed using Trimmomatic software (version 0.30) (Bolger, Lohse & Usadel, 2014) with parameters ILLUMINACLIP, SLIDINGWINDOW:4:30, MINLEN:36. Before assembly, reads were digitally normalized using Trinity submodule with parameter maximum coverage 50x. Trinity (version 2.1.0) software (Grabherr et al., 2011) was used for *de novo* transcriptome assembly of clean reads. CD-HIT-EST (version 4.6.1) (Fu et al., 2012) was employed for clustering of all five assemblies with parameter “-c 0.95”. CDS sequences for *T. aestivum* were obtained from the Ensembl database (Hubbard et al., 2002). Assembled *T. kiharae* contigs were mapped to *T. aestivum* set using BLASTN algorithm (version 2.2.25+) (Altschul et al., 1990) with parameter *e*-value = 1 × 10^−6^. Contigs without BLAST hits were annotated by GeneMarkS-T (Tang, Lomsadze & Borodovsky, 2015) with default parameters. For quality evaluation of transcriptome assembly, BUSCO (version 1.2) software (Simão et al., 2015) was used with default parameters. This software compares contigs of assembly with single-copy orthologs.

### Identification of DEFLs in wheat transcriptomes

**Figure 1:**
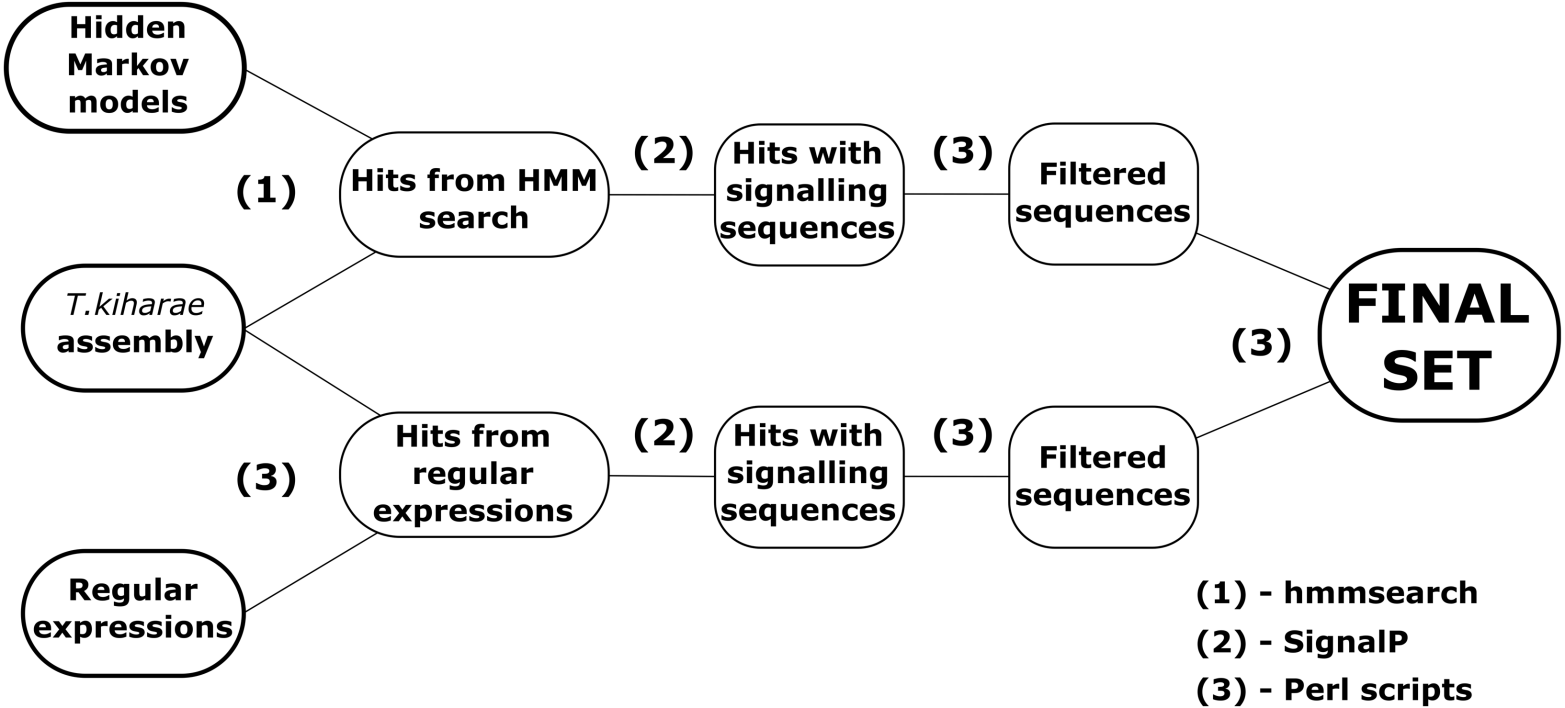
A workflow for DEFL identification in *T. kiharae* transcriptomes.

Two pipelines for revealing DEFLs in transcriptome data were developed in Perl (Fig. 1). Both pipelines can be run in Linux command line and at the input accept files with amino acid sequences in FASTA format.

The first pipeline is based on the method of hidden Markov models. The ready models of DEFL precursors were obtained from SPADA (Zhou et al., 2013). These models describe sequences that belong to one of four groups of motifs presented in Table 1. The models were created for amino acid sequences of defensins and defensin-like peptides detected in *Arabidopsis thaliana* and *Oryza sativa* by Silverstein et al. (2007). The pipeline works in several steps. First, the hidden Markov models are aligned against the transcriptome with hmmsearch from HMMER package (Durbin et al., 1998). Amino acid sequences in FASTA-like formats are required as input. Next, the detected sequences are filtered by the Perl scripts. The first script filters the hits by *E*-value (*E*-value <10^−3^). The second script detects signal peptides in the remaining sequences of defensin/DEFL precursors. It uses console version of SignalP v4.1 for this purpose (Bendtsen et al., 2004). Sequences without signal peptides are discarded. After that, the third script checks the discovered sequences to match the structure MZ..Z{C}m{X}n{C}l{X}k..*, where MZ..Z is a signal peptide; M, methionine; Z, any amino acid; C, cysteine; X, any amino acid residue except cysteine; m,n,l, *k* = 1,2,3…; *is a stop codon. Thus, the resulting precursor of a defensin-like peptide consists of a signal peptide that starts with methionine and a cysteine motif. After all quality control processes, the nucleotide sequences of peptides are detected by a specific script written in Perl. It uses the coordinates of the discovered precursors in amino acid sequences of transcriptome contigs and counts the corresponding coordinates in nucleotide sequences of the original nucleotide sequence of the translated contig. As a result, a collection of predicted amino acid and nucleotide sequences of identified defensin-like precursors is obtained.

**Table 1:** A list of defensin cysteine motifs used for DEFL mining in wheat transcriptomes.

**Motif**	**Approximate length (aa)**
C-X{4,25}-C-X{2,12}-C-X{3,4}-C-X{3,17}-C-X{4,32}-C-X-C-X{1,6}-C	40–70
C-X{3,21}-C-X{2,12}-C-X{3,4}-C-X{3,15}-C-X{4,23}-C-C-C	40–60
C-X{2,14}-C-X{3,5}-C-X{3,16}-C-X{4,28}-C-X-C	25–50
C-X{3,5}-C-X{8,17}-C-X{4,6}-C	20–30

**Notes.**

Amino acids are designated as follows: C, cysteine, X, any amino acid residue except cysteine. In curly brackets, a range for a variable number of X-type amino acids is indicated.

The second pipeline uses the method of regular expressions to detect sequences of putative defensin/DEFL precursors. This pipeline consists of scripts that scan transcriptome for sequences that match certain regular expressions. The general structure of regular expressions was as shown above. They include known structures of cysteine motifs found in defensin-like peptides (Table 1). The structure of regular expressions also considers the methionine residue at the beginning of the sequence. After obtaining a set of sequences that satisfy the structure of constructed regular expressions, the identified sequences are filtered by the presence of a signal peptide. At this step, the script from the first pipeline with the corresponding function is used. Finally, the nucleotide sequences are obtained using the same approach as above.

The resulting set of sequences is compared to the one obtained by hidden Markov models. Thus, using two pipelines, a more complete set of putative defensin/DEFL sequences is generated.

All identified putative defensin-like peptides were tested by CS-AMPPred program to predict if they belong to antimicrobial peptides (Porto, Pires & Franco, 2012). Domain identification was performed using InterProScan (Quevillon et al., 2005). Isoelectric point (pI) for each putative mature defensin was calculated by IPC tool (Kozlowski, 2016). All alignments and phylogenetic trees were constructed using Vector NTI Advance 9 software.

### Differential DEFL gene expression analysis

Differential DEFL gene expression analysis was based on read counts from infected, elicitor-treated, and pretreated with the elicitor and infected seedlings compared to those obtained from untreated control seedlings. To estimate DEFL gene expression levels, reads from four libraries were mapped to the final assembly produced by combining all libraries using bowtie2 software with default parameters. Raw read counts were obtained by samtools idxstats (Li et al., 2009). Expression values for individual DEFL coding sequences were calculated as Counts per Million Mapped Reads (CPM). Minimal expression threshold was defined as the minimal value of the maximal CPM value of predicted DEFL in four libraries. Differentially expressed genes (DEGs) were those with an expression fold change ≥2 (up-regulation) or ≤0.5 (down-regulation).

DEFL gene expression patterns were represented by heat maps (R package gplots v3.0.1).

### RT-PCR validation

A total of 3 µg of total RNA obtained by combining RNA preparations from all four samples were used for rapid amplification of cDNA ends using the Mint kit (Evrogen, Moscow, Russia) according to the manufacturer’s instructions. The amplified cDNAs coding for specific DEFLs were synthesized using high-fidelity Tersus DNA polymerase (Evrogen, Moscow, Russia) and gene-specific primers (Table S1). PCR conditions were as follows: initial denaturation step at 94 °C for 2 min followed by 30 cycles of denaturation at 94 °C for 30 s, primer annealing at 52 °C–63 °C for 30 s, and primer extension at 72 °C for 30 s, with the final extension of 5 min at 72 °C. The amplified fragments were separated by agarose gel electrophoresis and isolated from the gel with the Cleanup Standard kit (Evrogen, Moscow, Russia). All purified PCR fragments were sequenced on an ABI PRISM 3730 instrument (Applied Biosystems, Foster City, CA, USA).

## Results

### Protection of *T. kiharae* seedlings from *F. oxysporum* infection by elicitor metabolites from *F. sambucinum* strain FS-94

First, we checked whether the elicitor metabolites of *F. sambucinum* strain FS-94 protect *T. kiharae* seedlings from *F. oxysporum* infection. Figure S1 shows that seed treatment with *F. sambucinum* metabolites abolished disease symptom development in most *T. kiharae* seedlings, as compared to *F. oxysporum*-infected seedlings. Of 50 pretreated seedlings, none displayed root rot symptoms, while inoculation of seeds with *F. oxysporum* without pretreatment with the elicitors resulted in 80% death of seedlings. Thus, the elicitors from *F. sambucinum* indeed protect wheat seedlings from *F. oxysporum* infection.

### *De novo* Assembly of wheat transcriptomes

Sequencing of four cDNA libraries produced 59167054 raw reads for the control sample (Cont), 53657084 reads for infected (Inf), 67443211 reads for elicitor-treated (Ind), and 61440845 reads for IR-displaying sample (IR). *De novo* assembly of clean reads was performed using Trinity software (version 2.1.0) separately for each sample and for a better representation of transcripts, after combining all samples. Statistics of transcriptome assemblies are shown in Table S2. The quality of assemblies was assessed by BUSCO, which is based on evaluation of fully assembled single-copy conservative orthologs for higher plants. The results of this analysis are presented in Table S3. A small number of missing single-copy orthologs (missing BUSCOs) indicated that the quality of assemblies was sufficient for further analysis. After that, all five assemblies were merged into a single assembly. The resulting assembly was clustered using cd-hit-est software. A set of 295768 sequences was obtained after clustering. This value exceeds considerably the number of genes predicted for *T. aestivum* genome (from 101,000 to 120,000 genes). To filter false positives, assembled *T kiharae* sequences were mapped to *T. aestivum* genomic sequences by BLAST (version 2.2.25+), and assembly contigs without hits were excluded. 168,021 sequences remained in the resulting contig set, which were annotated using GeneMarkS-T. Sequences without predicted ORFs were also excluded from the data set. After all filtering stages, a final assembly consisting of 127,707 contigs was obtained.

### Identification of DEFLs in wheat transcriptomes

In this work, we used two approaches to predict DEFLs in wheat transcriptomes. One is based on hidden Markov models, and the other uses the method of regular expressions (Fig. 1). As a result, 143 DEFLs were predicted in wheat. Identified DEFLs conformed to the requirements obligatory for plant AMPs: the presence of methionine at the beginning and stop codon at the end of the precursor protein, and the presence of a signal peptide defined by SignalP.

According to the number of cysteine residues and their arrangement in the mature peptide domain, identified DEFLs were classified into ten groups: four main groups as specified by Silverstein et al. (2007) – two groups with eight cysteines, one group with six cysteines, and one group with four cysteines, and DEFLs with additional cysteine residues were assigned to groups 5–10.

#### Group 1 DEFLs

Group 1 DEFLs encode precursors of classical defensins with the 8-cysteine motif as follows: CX{4,25}CX{2,12}CX{3,4}CX{3,17}CX{4,32}CXCX{1,6}C, where C is cysteine, X is any other amino acid, and the numbers in brackets show the range of residues between neighboring cysteines. Group 1 is the most abundant DEFL group with 52 members named from DEFL1-1 to DEFL1-52 (Table S4). Group 1 DEFLs consist of a signal peptide and a mature peptide domain, therefore, encode class 1 defensins. Three DEFLs (DEFLs 1-13, 1-30 and 1-42) have short C-terminal extensions (Table S4). The length of the signal peptide in the precursors varies from 20 to 47. The length of the mature peptide is in the range from 46 to 60. Predicted mature defensins are basic peptides with pI from 7.23 to 9.93.

BLAST search in the NCBI database showed that 88.5% of DEFLs belonging to this group show sequence similarity to earlier isolated *T. kiharae* defensins, and defensins of *T. aestivum*, *T. turgidum*, *T. urartu*, *Aegilops tauschii* and *Hordeum vulgare*. The identity score varies from 61 to 99%. Two sequences DEFL1-15 and DEFL1-16 were identical to the peptides from the database XP_020164981.1 and BAK07823.1 at the amino acid level, but differed at the nucleotide level with 99 and 98% nucleotide identity, respectively. Only one sequence DEFL1-23 was 100% identical to *Ae. tauschii* peptide (XP_020172227.1) both at the nucleotide and amino acid levels. Solely 11.5% of discovered DEFLs were annotated as “hypothetical” or “predicted” proteins. However, their cysteine motifs, as well as their amino acid sequences show similarity to typical defensins (see below). A check with the CS-AMPPred program showed that all these peptides satisfied the criteria for AMPs. Thus, all except one discovered DEFL1-23 in group 1 DEFLs represent novel genes.

The phylogenetic tree based on DEFL sequences was built using Vector NTI package (Fig. S2). Six main subgroups (A–F) comprising varying number of DEFLs could be discriminated: subgroups A and D with only two members, subgroup B with 16, subgroup C with 19, and subgroups E and F with 6 DEFLs. DEFL1-17 was not assigned to any subgroup.

**Figure 2:**
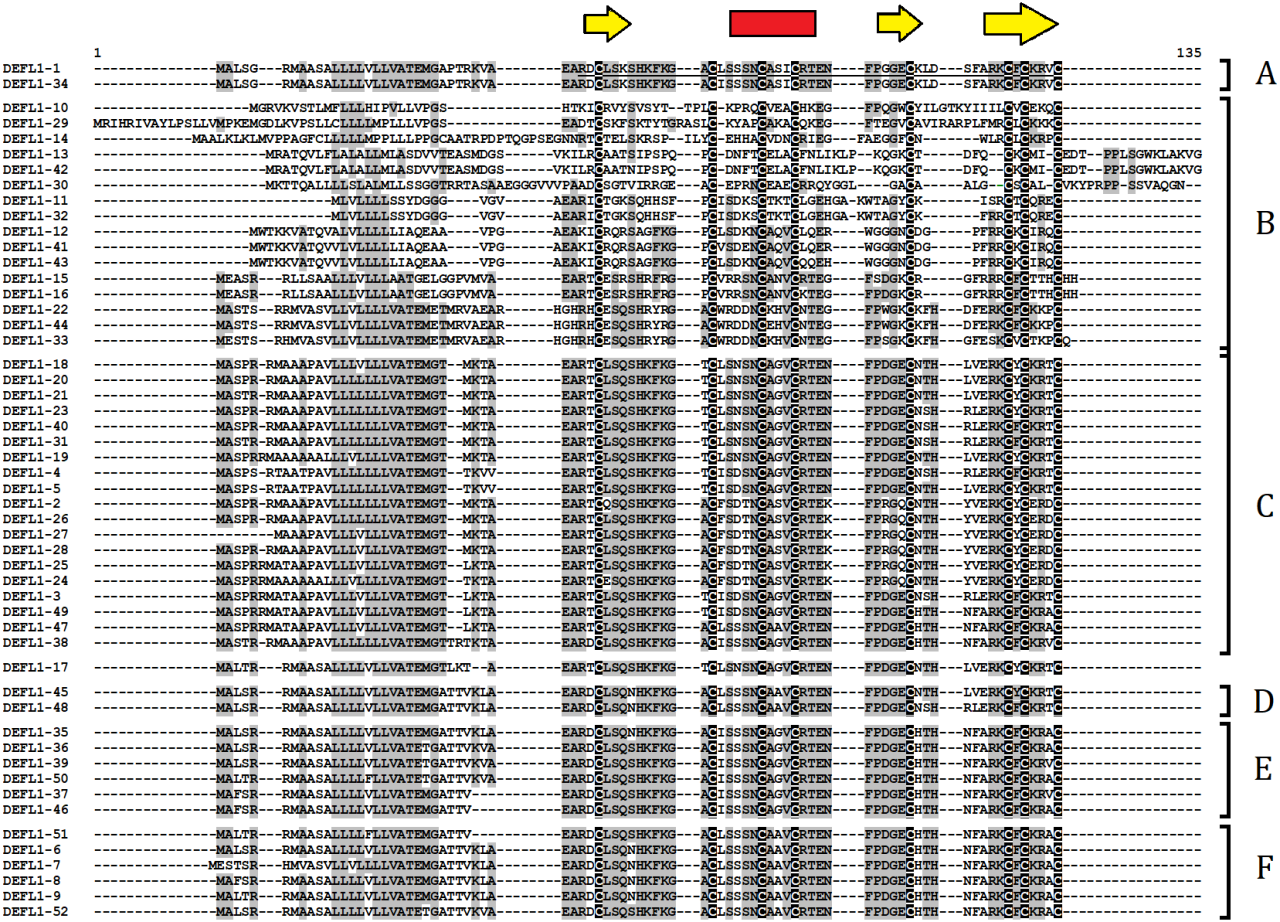
Multiple sequence alignment of *T. kiharae* group 1 DEFLs. Cysteine residues are shaded black, while identical amino acids are shaded gray. The positions of β-strands (yellow) and the α-helix (red) are indicated above the figure. Subgroups of similar DEFLs designated A–F are shown to the right of the alignment.

As it is seen from multiple sequence alignment (Fig. 2), sequence variation is the highest in subgroup B. All other subgroups are comprised of highly similar polypeptides. High sequence similarity is observed both in the signal peptide region and the mature defensin domain. Some sequences in each subgroup are virtually identical and differ in a single amino acid residue (see, for example DEFLs 1-1 and 1-34 in subgroup A and DEFLs 1-2 and 1-26 from subgroup C). Some precursors differ only in the signal peptide region, while the mature peptides are identical. These DEFLs are colored similarly in Table S4.

To compare *T. kiharae* group 1 DEFL-derived defensins with those of other cereals, a phylogenetic tree of *T. kiharae* predicted mature defensins, *T kiharae* seed defensins and selected defensins of other cereals was constructed (Fig. S3). Defensins of *Leymus arenarius* discovered earlier by whole-transcriptome analysis were also included in comparison (Slavokhotova, Shelenkov & Odintsova, 2015). In the phylogenetic tree, six subgroups (A–F) could be distinguished. DEFL-derived defensins are distributed among all six subgroups, while *T. kiharae* seed defensins (Tk-AMP-D) are clustered into two subgroups. Multiple sequence alignment of defensins belonging to each subgroup is shown in Fig. S4.

All group 1 DEFLs were checked with the CS-AMPPred program, and all of them were predicted to possess antimicrobial activity. Domain analysis with InterProScan showed that all group 1 DEFLs belong to gamma-thionin (defensin) family (Table S5).

#### Group 2 DEFLs

**Figure 3:**
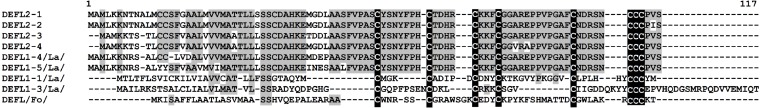
Multiple sequence alignment of *T. kiharae* group 2 DEFLs. DEFLs from *L. arenarius* (La) (Slavokhotova, Shelenkov & Odintsova, 2015) and *F. oxysporum* (Fo) (GeneBank EXK23984.1) were included for comparison. Cysteine residues are shaded black, identical amino acids are shaded gray.

Group 2 DEFLs possess the motif CX{3,21}CX{2,12}CX{3,4}CX{3,15}CX{4,23}CCC in the mature peptide region. Four precursor sequences (DEFL2-1 – DEFL2-4) with this cysteine motif were retrieved from the transcriptome data (Table S4). Two of them (DEFL2-3 and DEFL2-4) had a homologue in *T. urartu* with high identity score (97 and 99% for DEFL2-3 and DEFL2-4, respectively) annotated as “a hypothetical protein”. The other two DEFLs were similar to a predicted protein from *H. vulgare* with identity of 82 and 80%, for DEFL2-1 and DEFL2-2, respectively. In contrast to group 1 defensins, these DEFLs are neutral, pI of predicted mature peptides varies from 6.37 to 7.10. Amino acid sequence alignment of predicted DEFLs belonging to this group is shown in Fig. 3. DEFLs from a wild cereal *L. arenarius* identified earlier were included for comparison. As it is seen from this figure, *T. kiharae* group 2 DEFLs form a family of closely related peptides. The peptides DEFL2-1 and DEFL2-2 differ in a single amino acid residue, and the peptides DEFL2-3 and DEFL2-4 vary at two positions in the predicted mature peptide region. It is of particular interest that peptides with the same cysteine motif were discovered earlier in *L. arenarius* using the whole-transcriptome sequencing approach (Slavokhotova, Shelenkov & Odintsova, 2015). Two *L. arenarius* DEFLs – DEFL1-4 and DEFL1-5 are virtually identical to *T. kiharae* peptides in the putative mature defensin domain (Fig. 3). This sequence conservation between two cereal species points to important common functions. It deserves noting that in the wheat transcriptomes, a sequence with the same 8-Cys motif comprising three consecutive cysteines that showed 100% similarity with a hypothetical protein from *F. oxysporum* f. sp. *melonis* was discovered (DEFL(Fo) in Table S4). We suppose that it originated from the fungus used for inoculation of *T. kiharae* seedlings. However, the amino acid sequence of this fungal DEFL differed considerably both from those of wheat and *L. arenarius* (Fig. 3). Thus, this type of DEFLs is found not only in plants, but in fungi as well. To predict the antimicrobial activity of group 2 DEFLs, we used the CS-AMPPred program. According to this program, all group 2 DEFLs are AMPs.

#### Group 3 DEFLs

**Figure 4:**
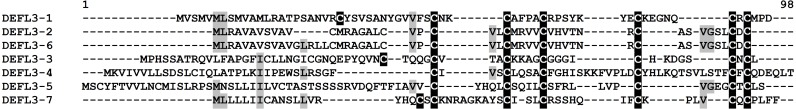
Multiple sequence alignment of *T. kiharae* group 3 DEFLs. Cysteine residues are shaded black, and identical amino acids are shaded gray.

Group 3 DEFLs are characterized by the presence of six cysteines arranged in the motif CX{2,14}CX{3,5}CX{3,16}CX{4,28}CXC. Seven sequences retrieved from the wheat transcriptome data conformed to this requirement: four of them had only this 6-Cys motif, while three other had an additional cysteine residue before the motif (Table S4). These DEFLs had no hits in the NCBI database, therefore, they are new proteins. All of predicted mature peptides are neutral, except for DEFL3-7, which is a basic peptide. According to the CS-AMPPred program, two of group 3 DEFLs (DEFL3-4 and DEFL3-5) were not AMPs, while the remaining DEFLs were assigned to AMPs. All except one member (DEFL3-1) produced no hits with known protein domains during InterProScan analysis (Table S5). It is of particular interest that the InterProScan program revealed a RING-type zinc finger domain in DEFL3-1. Znf motifs occur in different proteins and represent stable scaffolds that have evolved specific functions (IPR013083). They were shown to bind proteins, nucleic acids (RNA and/or DNA) and lipids. We may speculate that this domain has evolved in some AMPs to provide efficient binding to their microbial targets, either proteins, or nucleic acids and lipid molecules. Amino acid sequence alignment of group 3 DEFLs is shown in Fig. 4. As seen from this figure, two DEFLs (DEFL3-2 and DEFL3-6) are highly similar peptides with sequence variation restricted to the predicted signal peptide region. All other group 3 DEFLs show extreme sequence variation except for the position of cysteine residues.

#### Group 4 DEFLs

**Figure 5:**
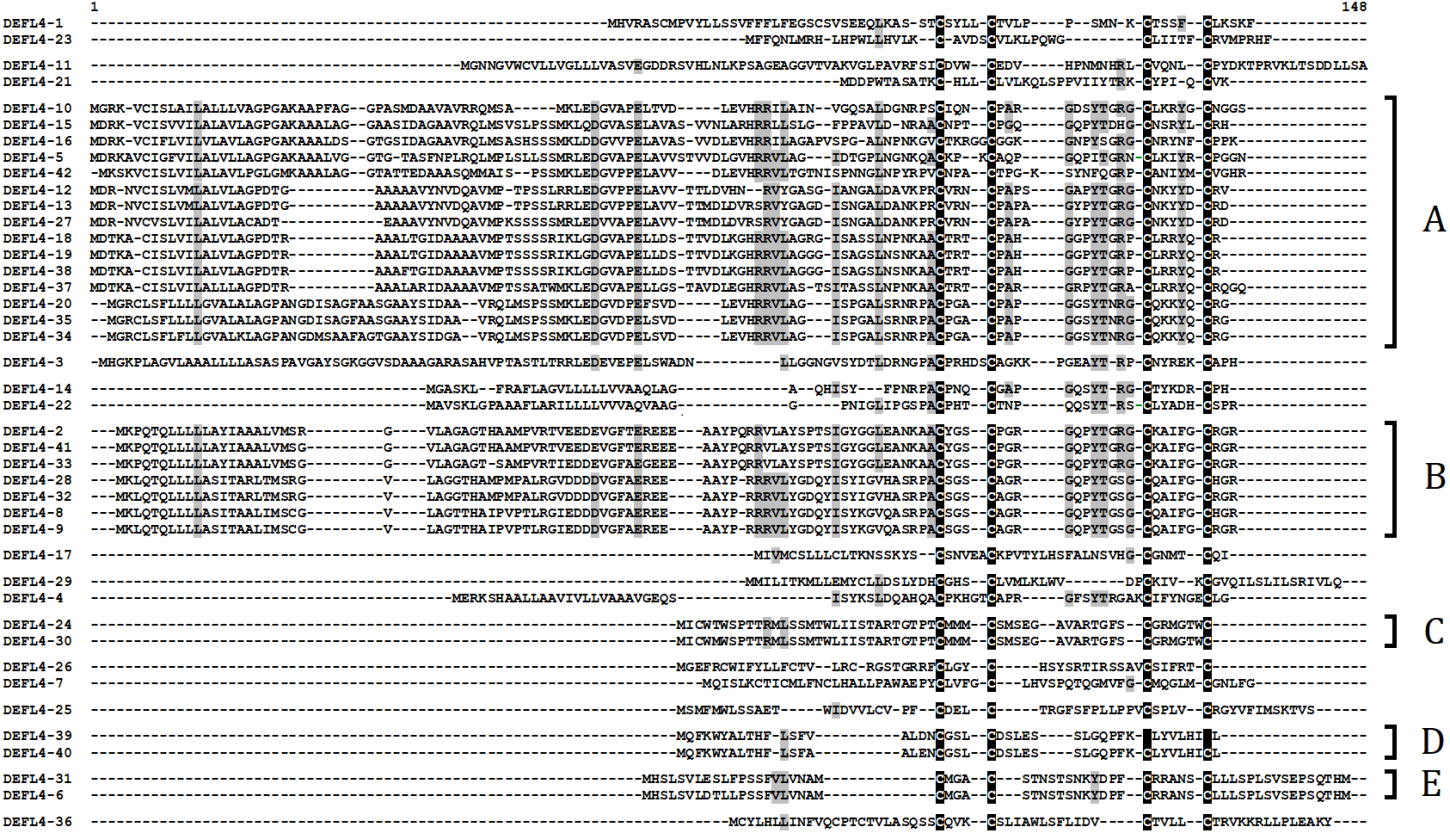
Multiple sequence alignment of group 4 DEFLs. Cysteine residues are shaded black, identical amino acids are shaded gray. Subgroups of closely related DEFLs designated A–E are indicated to the right of the alignment.

Group 4 DEFLs comprises 42 members (Table S4). They are characterized by the presence of a 4-Cys motif as follows: CX{3,5}CX{8,17}CX{4,6}C. This group is rather heterogenous and includes both acidic and basic polypeptides, with pI ranging from 4.08 to 10.11. 24 DEFLs (57%) are annotated as “uncharacterized proteins” from the polyploid wheat closest relatives – *Ae. tauschii* and *T. urartu*. The remaining 17 DEFLs had no BLAST hits, and are likely products of new *T. kiharae* genes. One sequence DEFL4-14 showed similarity to a “predicted protein” from *H. vulgare* (56% identity). One DEFL4-20 was 100% identical at the amino acid level to the “uncharacterized protein” of *Ae. tauschii* (XP_020197492.1), and one DEFL4-42 showed 100% identity both at the amino acid and nucleotide levels to the “unnamed protein product” from *T. aestivum* (CDM86531.1). It is interesting that sequence analysis for characteristic domains with InterProScan revealed that four group 4 DEFLs (DEFLs 4-16, 4-20, 4-34, and 4-35) belong actually to RALFs, small, cysteine-rich peptides involved in plant growth and development (Pearce et al., 2001) (Table S5). It is not surprising because Silverstein’s cysteine motifs postulated for 4-cysteine DEFLs overlap with those for RALFs (Silverstein et al., 2007). RALFs are synthesized as preproproteins containing an N-terminal signal peptide that is necessary for secretion and a C-terminal mature peptide with four cysteines forming two disulfide bonds. It should be noted that true RALFs possess two short conserved sequences that are important for their activity: the RRXL cleavage site and the YISY motif required for receptor binding (Campbell & Turner, 2017). All four DEFLs assigned to RALFs by InterProScan possess only the RRXL motif required for proteolytic processing of the precursor, while the second conserved YISY motif is missing, therefore, it seems more reasonable to describe these wheat peptides as RALF-related. Of group 4 DEFLs, 29 (69%) were predicted to be AMPs. The phylogenetic tree based on precursor sequences (Fig. S5) separated these DEFLS according to sequence similarity into two large subgroups A and B with 15 and 7 members, respectively (Fig. 5), and three pairs of extremely similar DEFLs that differ in 1-3 amino acid residues (subgroups C, D, and F) (DEFL4-24 – DEFL4-30, DEFL4-39 – DEFL4-40, and DEFL4-31 – DEFL4-6). Other DEFLs in this 4-Cys group have low sequence similarity to the above-mentioned subgroups, although the characteristic cysteine motif is well conserved.

#### Groups 5-10 DEFLs

In addition to DEFLs with a single 4-Cys motif in the predicted mature peptide region, peptides with additional cysteines (from 1 to 7) were discovered (Table S4). They were referred to as DEFL groups 5–10. 27 DEFLs (71%) had no significant BLAST hits in the NCBI database, therefore, they are encoded by novel *T. kiharae* genes, 11 DEFLs (29%) were annotated as “hypothetical” or “uncharacterized” proteins of *Ae. tauschii* and *T. urartu* with high sequence identity score (74–99%). Two DEFLs (DEFL9-4 and DEFL10-6) were identical to uncharacterized proteins of *Ae. tauschii* (XP_020175127.1 and XP_020179991.1, respectively) at the amino acid level, and the latter peptide, both at the amino acid and nucleotide levels. Among DEFLs of these groups, acidic (e.g., DEFL6-1) and basic (e.g., DEFL5-9) peptides were found. 16 DEFLs (42%) were predicted to possess antimicrobial activity. All members of these groups produced no hits with known protein domains during InterProScan analysis (Table S5). However, in DEFL5-8, InterProScan identified signatures characteristic of endonuclease/exonuclease/phosphatase family, which are possibly related to specific functions of this non-AMP DEFL.

**Figure 6:**
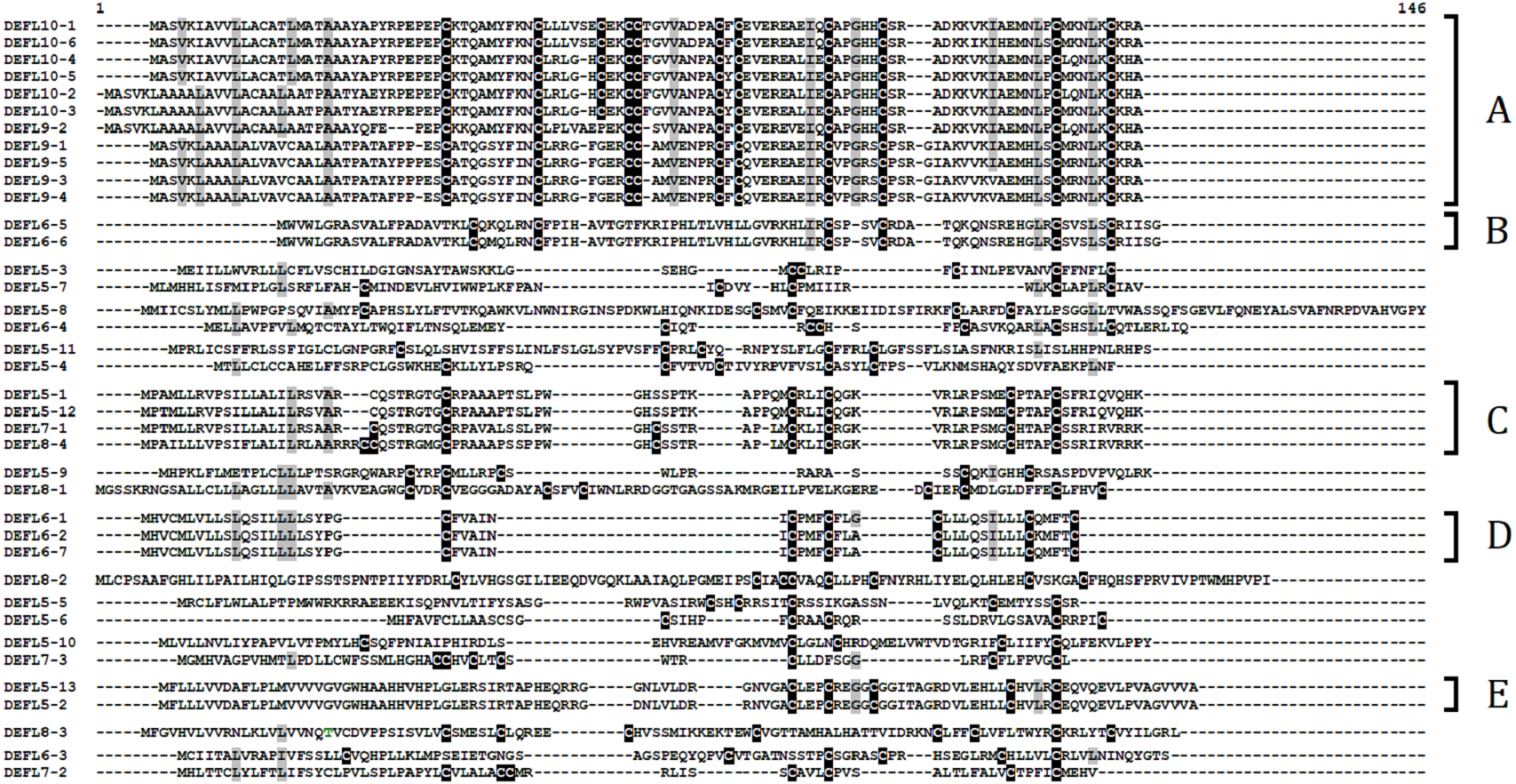
Multiple sequence alignment of *T. kiharae* DEFLs belonging to groups 5–10. Cysteine residues are shaded black, identical amino acids are shaded gray. Subgroups of closely related DEFLs designated A–E are indicated to the right of the alignment.

The phylogenetic tree of DEFL groups 5–10 precursor sequences is shown in Fig. S6. Sequence alignment of DEFLs belonging to groups 5–10 is presented in Fig. 6. Several subgroups of related peptides can be isolated. 11 DEFLs (subgroup A in Fig. 6) form a group of closely related peptides, although the number of cysteines in the predicted mature peptide domain varies (10 or 11). Subgroup B is represented by two highly similar peptides DEFL6-5 and DEFL6-6. The same holds true for subgroup D consisting of 3 DEFLs. Subgroup C also comprises four closely related peptides with different number of cysteine residues (5–8). Subgroup E combines two highly similar peptides DEFL5-13 and DEFL5-2. Other DEFLs in this group show low sequence similarity with each other.

### Validation of DEFL gene expression by RT-PCR

To confirm expression of predicted DEFLs, 24 DEFL transcripts belonging to different structural groups were selected. Total wheat RNA was reverse transcribed, and PCR with specific primers (Table S1) was performed. All obtained sequences were identical to those generated by Illumina sequencing. Thus, the sequences of selected DEFL genes were confirmed.

### Differentially expressed DEFL genes in wheat transcriptomes

**Figure 7:**
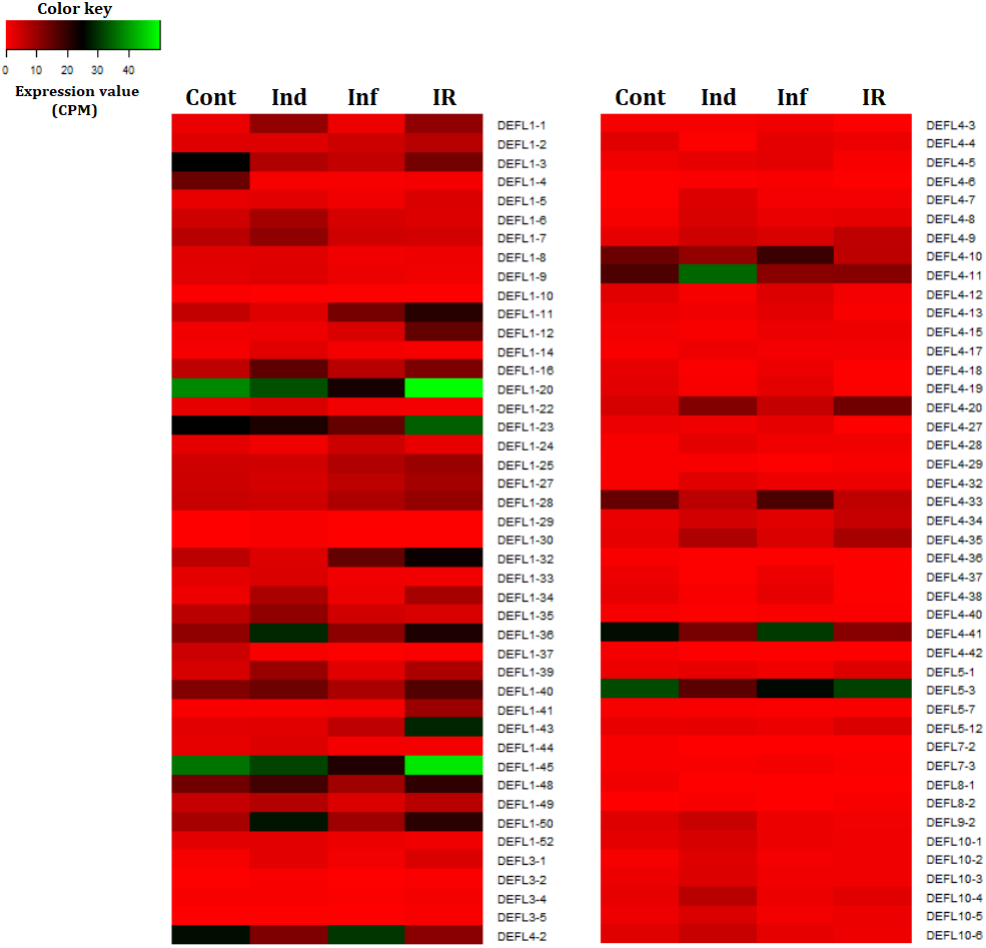
Heatmap of differentially expressed DEFL genes. Cont, Ind, Inf and IR, control, induced, infected and IR-displaying samples, respectively.

**Figure 8:**
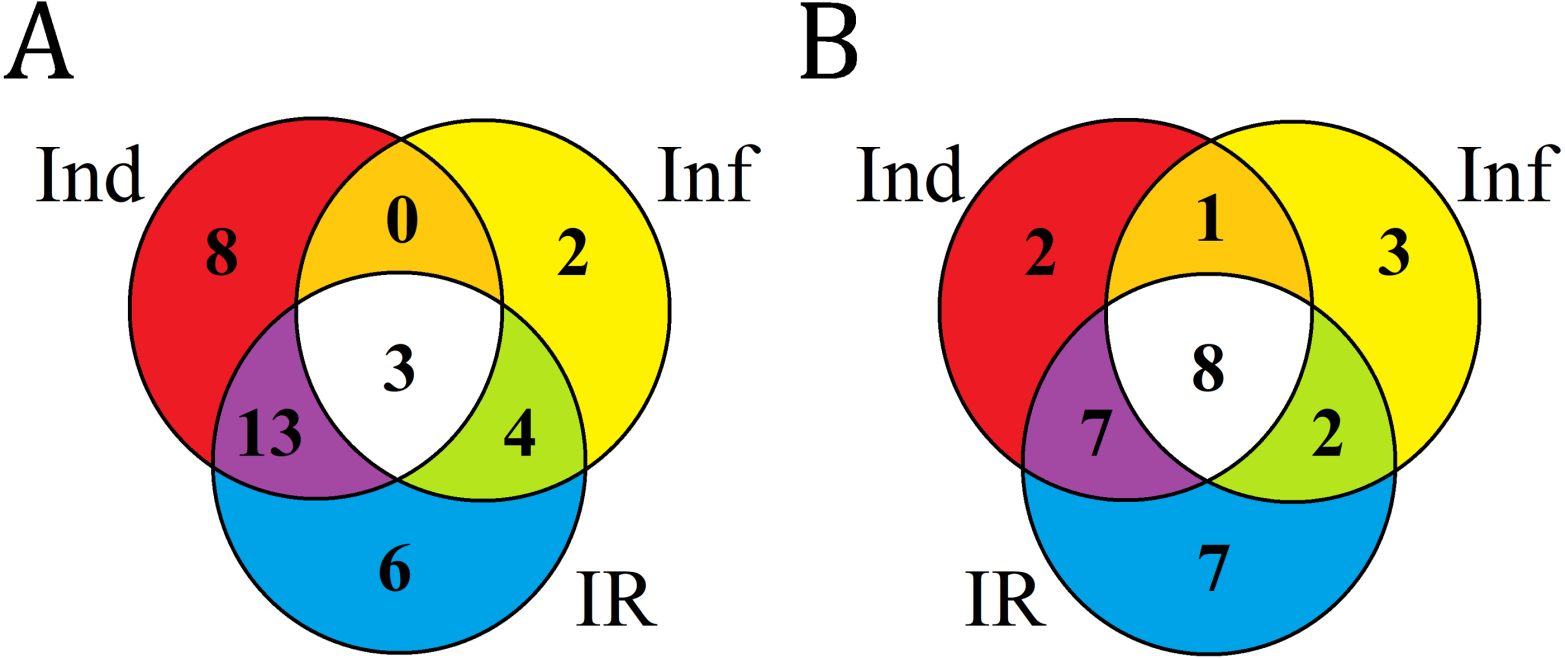
Venn diagram showing the number of DEFL genes up- or down- regulated compared with control in elicitor-treated (Ind), *F. oxysporum*-infected (Inf) and IR-displaying *T. kiharae* seedlings. (A) Up-regulated DEFL genes. (B) Down-regulated DEFL genes. For up-regulated DEFL genes, expression fold change was ≥2, for down-regulated, ≤0.5.

We compared expression levels of different DEFL genes in four transcriptomes (Fig. 7). As it is seen from Figs. 7 and 8, different states of wheat seedlings are characterized by distinct patterns of DEFL expression. The expression level of a set of DEFL genes did not change more than two-fold in all four transcriptomes (e.g., DEFLs 1-15, 2-3, 2-4, 4-1, 4-22, 4-30, 4-31, 5-4, 5-5, 5-6, 6-1, 6-3). It is of particular interest that among these genes, DEFL4-1 occupies a special place: the expression level of this gene was at least twice as high in all four transcriptomes compared to other DEFL genes. Expression of DEFL4-1 was confirmed by RT-PCR. Meanwhile this DEFL has no BLAST hits and is evidently a novel *T. kiharae* gene. Further studies of the role of this gene in wheat plant physiology are therefore of particular importance. Expression profiling showed that in addition to DEFL genes irresponsive to all treatments, from 16% to 35% of DEFLs changed expression patterns depending on the treatment (elicitor, fungus, or elicitor + fungus) (Fig. S7).

#### Infection with *F. oxysporum*

In the transcriptome of infected seedlings (Inf/Cont in Table S6), 16% of genes were responsive to infection, more genes were down-regulated (60% of responsive DEFL genes, 14 DEFLs) than up-regulated (40%, 9 DEFLs) (Fig. S7). Most up-regulated genes encode groups 1 and 4 DEFLs, only one transcript encodes group 7 DEFL. Down-regulated genes were more diverse. Most of them again belonged to groups 1 and 4; however, single DEFLs from groups 3, 5, 7, and 8 were also down-regulated (Table S7).

#### Treatment with FS-94 metabolites

Comparison of the transcriptome of seedlings treated with *F. sambucinum* elicitors with that of control seedlings (Ind/Cont in Table S6) demonstrated that expression of more genes was affected: 57% of elicitor-responsive DEFLs (24 DEFLs) were up-regulated, and 43% (18 DEFLs) were down-regulated (Fig. S7). Most of up-regulated genes belonged to DEFL groups 1, 3, 4 and 10, single DEFL from group 8. Among down-regulated genes were four group 1 DEFLs, 11 group 4 DEFLs, and single DEFLs from groups 5, 7, and 8 (Table S7).

#### Pretreatment with the elicitors and infection with *F. oxysporum*

In IR-displaying seedlings (IR/Cont in Table S6) the portion of differentially expressed DEFL genes was the highest compared to elicitor-treated and infected seedlings: expression of 35% of genes changed in comparison with untreated control seedlings, of them 51% (26 DEFLs) were up-regulated and 49% (24 DEFLs) were down-regulated (Fig. S7). The majority of up-regulated genes belonged to groups 1 and 4, the remaining 3 DEFLs, to group 3 (Table S7). Of down-regulated genes, 67% (17 DEFLs) were group 4 DEFLs, 20% (5 DEFLs) were group 1 DEFLs (Table S7).

#### Comparison of three treatments

Analysis of composition of differentially expressed DEFL genes in infected (Inf/Cont), elicitor-treated (Ind/Cont) and IR-expressing (IR/Cont) seedlings compared to control untreated seedlings showed that they represent overlapping groups of genes (Fig. 8, Table S7).

Three groups of DEFL genes could be isolated: (1) DEFLs up-or down-regulated in all three transcriptomes; (2) in 2 transcriptomes; (3) only in one transcriptome. For up-regulated genes, 3 DEFL genes (DEFLs 4-7, 4-8, and 4-28) were induced under all conditions (Fig. 8A, Table S7). Eight DEFL genes were specifically induced by the elicitors, 2 by *F. oxysporum* infection, and 6 were responsive to infection only in IR-expressing seedlings. For down-regulated genes, 8 DEFLs (DEFLs 1-4, 1-10, 1-37, 4-36, 4-40, 4-42, 7-2, 8-1) were repressed in all three experimental set-ups, 2 DEFLs were specifically repressed by the elicitors, three by *F. oxysporum*, and seven were down-regulated only in IR-expressing seedlings (Fig. 8B).

Infection of elicitor-treated seedlings resulted in up-regulation of 22 DEFL genes compared to untreated infected seedlings (IR/Inf) (Fig. S8A, Table S8). Most of up-regulated genes belong to group 1 DEFLs, the remaining, to groups 3–6. Sixteen DEFL genes were down-regulated (Fig. S8B). Fourteen of them belong to group 4, one to group 1 and one to group 7 (Table S8).

In comparison with elicitor-treated seedlings (IR/Ind), in IR-displaying seedlings 11 DEFL genes were up-regulated (Fig. S8A, Table S8), and 26 DEFL genes were down-regulated (Fig. S8B, Table S8).

It is of particular interest that some DEFLs induced by the elicitor (DEFLs 1-14, 1-29, 1-30, 4-7, 4-11, 10-2, 10-4, 10-5 in Table S7) were down-regulated in IR-displaying plants (Table S8). Two explanations are possible: (1) these DEFLs are not needed for protection against *F. oxysporum*, or (2) they have already been synthesized in sufficient amounts in elicitor-treated plants, so that additional expression of the corresponding genes is not necessary.

Thus, wheat DEFL expression profiling showed that most variation in DEFL expression level was observed in group 1 and group 4 DEFLs, namely in classical defensins and 4-Cys-containing defensins (Fig. 7).

It deserves special attention that expression analysis of DEFLs encoding identical mature peptides showed that in some groups, the expression pattern of DEFLs was different (e.g., DEFLs 1-3 and 1-4), while in some groups (e.g., DEFLs 1-17 – 1-21), it was similar (Table S6).

## Discussion

Wheat is the crop of global importance in temperate climate, being the main source of food for people and feed for livestock. Analysis of wheat defensive compounds is, therefore, of prime importance for enhancing disease resistance and yields. However, the repertoire of DEFL genes in wheat has not been studied so far. In this work, we analyzed DEFL composition in the synthetic allopolyploid *T. kiharae*. This species is highly resistant to pathogens. Earlier, using a peptidomic approach we isolated 11 and sequenced 6 defensins from seeds of this species (Egorov et al., 2005). However, the efficiency of biochemical methods for new DEFL discovery lags behind novel high-throughput NGS technologies. In this work, to explore the inventory of DEFL genes in *T. kiharae*, for the first time, global RNA sequencing and *de novo* transcriptome assembly were performed. Several approaches for AMP mining in RNA-seq data were described (Cândido et al., 2014; Slavokhotova, Shelenkov & Odintsova, 2015; Porto, Pires & Franco, 2017). In this work, to search for DEFL transcripts, an approach combining the method of hidden Markov models with that of regular expressions was used. To study the role of defensins in IR, wheat seedlings were treated with the elicitor metabolites of *F. sambucinum*, which, as we showed earlier, did not possess fungitoxic activity (Shcherbakova et al., 2011), but instead, acted as elicitors of induced resistance in *T. aestivum* against multiple fungal pathogens (Shcherbakova et al., 2012). To shed light on the role of discovered DEFLs in IR, differential profiling of DEFL gene expression was performed in transcriptomes obtained from healthy, elicitor-treated, infected, and IR-expressing *T. kiharae* seedlings. It should be specifically noted that although analysis of global gene expression using NGS technologies is a powerful novel tool to study the interactions of plants with pathogenic and beneficial microorganisms (Knief, 2014), only single studies report transcriptional changes in AMP gene expression induced by pathogens and biocontrol agents (Slavokhotova et al., 2017; Mondragon-Palomino et al., 2017; Miranda et al., 2017; Luo et al., 2018). Thus, in the model plant *A. thaliana,* transcriptome analysis by microarray showed that the rhizobacterium *Pseudomonas fluorescens* WCS417, which induced systemic resistance in plants, locally in the roots caused changes in expression of 97 genes, however, no changes in expression level of about 8,000 genes were observed in leaves. Only after challenge with *P. syringae* pv. *tomato* DC3000, 81 gene was up-regulated in ISR-expressing plants, among them the defensin PDF1.2 (Verhagen et al., 2004). In the transcriptomic analysis using RNA-Seq of *Brassica juncea* var. *tumida* Tsen responses to *Plasmodiophora brassicae* primed by the biocontrol strain *Zhihengliuella aestuarii*, up-regulation of six PR-proteins (five thaumatin family proteins and one LTP) in ISR-expressing plants induced by the biocontrol agent *Z. aestuarii* was shown. However, no changes in defensin gene expression were reported in this study (Luo et al., 2018).

Using our pipeline of DEFL mining, as many as 143 DEFL transcripts were discovered in wheat transcriptomes. All DEFLs have either a CS αβ motif common to defensins (Cornet et al., 1995) or a γ-core motif characteristic to all Cys-rich AMPs (Yount & Yeaman, 2004). Thus, DEFL genes in wheat represent a multigene family. The vast majority of discovered *T. kiharae* DEFL genes represent novel genes. Only three DEFLs exhibited 100% similarity to the annotated genes in the databases. Sequence variation in discovered DEFLs is likely to be responsible for functional diversification of DEFLs. The discovery of a divergent DEFL multigene family in wheat is in accordance with previous data obtained for other plant species. Thus, Silverstein et al. (2007) using bioinformatics-based approaches identified 317 DEFL genes in *A. thaliana* genome and 93 DEFL genes in rice genome. Sixty-two DEFLs were reported in the maize genome (Li et al., 2014). Wu et al. (2016) identified nine DEFL genes in the brachypodium genome, 12 in rice, 20 in maize and 10 in sorghum. Thirthy-seven DEFL genes were identified in our study by RNA-seq in the wild cereal *L. arenarius* (Slavokhotova, Shelenkov & Odintsova, 2015), and twenty-four DEFL genes were discovered in the weed species *Stellaria media* belonging to the Caryophyllaceae family (Slavokhotova et al., 2017). Analysis of *M. truncatula* EST data revealed more than 300 nodule-specific DEFL genes (Fedorova et al., 2002; Mergaert et al., 2003; Graham et al., 2004). The number of discovered DEFLs in *T. kiharae* seedlings exceeds that detected in other cereals in genomic or EST data by bioinformatic techniques. The abundance of DEFL genes in *T. kiharae* is likely to be associated with the polyploid nature of this wheat species, with all three constituent genomes contributing DEFL genes to the polyploid.

We classified *T. kiharae* DEFLs identified in transcriptome data into 4 groups according to four cysteine motifs specified by Silverstein et al. (2007). DEFLs with additional cysteines were assigned to groups 5–10. In each group, subgroups of closely related peptides could be discriminated. Some sequences in each subgroup are virtually identical and differ in a single amino acid residue. Some DEFL genes encode identical defensins with variant signal peptides. Sequence similarity within subgroups of wheat DEFLs points to a significant role of duplications in the evolution of DEFL gene family, with high level of nucleotide identity observed in some instances indicating relatively recent duplications. These findings are consistent with the conclusions made by Silverstein et al. (2005) during the analysis of DEFL gene family in *A. thaliana* that evolution of DEFL genes occurred by successive rounds of tandem and segmental duplication followed by purifying or diversifying selection.

The classical defensins with the 8-Cys motif: CX{4,25}CX{2,12}CX{3,4}CX{3,17}CX{4, 32}CXCX{1,6}C represent the most numerous group 1 of *T. kiharae* DEFLs. Most DEFLs in this group show high sequence similarity and even identity at the amino acid level in the mature peptide region to defensins of *Ae. tauschii* and *T. urartu* (Fig. S4). This is not surprising since these diploid species contributed their genomes (D and A, respectively) to the polyploid wheat genomes in the course of evolution.

It is worth noting that although *T. kiharae* group 1 DEFLs show high sequence similarity to defensins earlier isolated from seeds of this species (Egorov et al., 2005), seed and seedling defensins represent different sets of peptides, confirming tissue-specific pattern of DEFL gene expression.

Comparison of classical (group 1) DEFLs with defensins from other cereals reveals groups of closely related peptides (Fig. S4). Of particular interest are defensins conserved among different Poaceae genera. For example, the mature defensin in *T. kiharae* DEFL1-16 is 100% identical to a defensin from *H. vulgare* (BAC07823.1) and DEFL3-2 from *L. arenarius*. Such conservation of sequences points to their ancient origin in the common ancestor before the divergence of the genera *Triticum*, *Hordeum* and *Leymus* and highlights the vital immune functions of these molecules for grasses.

In *T. kiharae* transcriptomes we discovered 4-Cys containing DEFLs with a characteristic cysteine motif CX{3,5}CX{8,17}CX{4,6}C which we named group 4 DEFLs. It should be specifically emphasized that DEFLs with this cysteine motif were earlier discovered only in nodules of the galegoid legumes. They were shown to possess antimicrobial activity to protect nodules from the pathogens (Mikuláss et al., 2016) and supposed to play a role in bacterioid differentiation (Mergaert et al., 2006). We showed that DEFLs with the same motif were well represented in wheat. We discovered as many as 42 DEFLs with this motif in wheat seedlings; furthermore, 38 DEFLs with this motif and additional cysteine residues in the mature peptide domain were also found in wheat (groups 5–10). In addition, changes in expression of group 4 DEFL genes were shown upon treatment with *F. sambucinum* elicitors and pathogen infection, as well as in IR-expressing seedlings indicating their involvement in stress response and IR.

We did not check the antimicrobial activity of identified putative DEFLs. However, the predicted defensins from group 1 DEFLs most likely possess antimicrobial activity. Two lines of evidence support this suggestion. Firstly, they show high sequence similarity to defensins with proven antimicrobial activity (Fig. S4). Thus, our antimicrobial assays of Tk-AMP-D1 defensin from wheat seeds showed that it inhibited growth of *F. graminearum* and *F. verticilloides,* while Tk-AMP-D6 was active against *F. verticilloides* (Odintsova et al., 2008). A close relative of *T. kiharae* DEFL1-17, *T. aestivum* defensin TAD1 induced during cold acclimation in winter wheat suppressed growth of the snow mold fungus *Typhula ishikariensis* and *F. graminearum*, the causal agent of head blight (Sasaki et al., 2016). Defensins from the weed *Echinochloa crus-galli* displayed potent antifungal activity against *F. graminearum*, *F. verticilloides* and *F. oxysporum* (Odintsova et al., 2008). The corn defensin PDC1 inhibited growth of *F. graminearum* (Kant, Liu & Pauls, 2009). The rice defensins DEF7 and DEF8 were effective against the bacteria *Xanthomonas oryzae* and *X. campestris* and the fungus *F. oxysporum* (Tantong et al., 2016; Weerawanich et al., 2018). Secondly, all group 1 DEFLs were checked with the CS-AMPPred program, and all of them were predicted to possess antimicrobial activity. However, to confirm antimicrobial activity, direct tests with isolated or recombinant defensins are nevertheless necessary. In addition to antifungal activity, on the basis of sequence similarity with cereal defensins that were shown to inhibit protein synthesis or insect α-amylases (Fig. S4), we may suggest the same functions for some wheat DEFLs. For example, subgroup B in Fig. S4 comprises defensins that inhibit protein synthesis, *α*-amylases and sodium channels. We may suggest similar functions to defensins derived from DEFLs 1-12, 1-40, 1-41, and 1-43. ZmES1 from subgroup D inhibits protein synthesis. So, defensins of DEFLs 1-10, 1-13, 1-14, 1-29, 1-30, 1-42 may possess the same function, although sequence similarity within this subgroup is rather low. Thus, on the basis of structure similarity with defensins, whose functions were established, we may hypothesize diverse functions for discovered wheat DEFLs: antifungal, antibacterial, inhibition of insect α-amylases and protein synthesis.

In order to elucidate the role of DEFLs in the phenomenon of induced resistance, we performed differential expression profiling of DEFL genes in wheat seedlings infected with *F. oxysporum*, treated with FS-94 elicitors, pretreated with the elicitors and infected with the fungus (IR-displaying). Along with DEFL genes whose expression level did not change more than two-fold in all experimental set-ups, a portion of DEFL genes displayed differential expression pattern. We suppose that wheat DEFL genes irresponsive to treatments might not be involved in immune response, but in some other functions.

We discovered that infection with *F. oxysporum* changed expression of about 16% of DEFL genes in wheat. Among them, more DEFL genes were down-regulated than up-regulated. It is noteworthy that during fungal infection, genes encoding DEFLs belonging to the same subgroup of similar peptides could display different expression pattern. For example, in subgroup B of group 1 DEFLs 1-11 and 1-12 were up-regulated, while DEFL1-22 was down-regulated. Up-regulation of DEFL genes may reflect activation of defense reactions in response to the pathogen, while down-regulation might manifest suppression of the immune response by the pathogen. It is of particular interest that suppression of DEFL gene expression in the weed *S. media* infected with the same fungus was observed in our previous work (Slavokhotova et al., 2017). Similar results were obtained by Mondragon-Palomino et al. (2017) on *Arabidopsis* species infected with F. *graminearum*: the same portion as in wheat (about 16% of all DEFLs) of DEFL genes was differentially expressed in response to *F. graminearum* infection, with down-regulated genes representing the largest proportion of differentially expressed genes. Taken together, down-regulation of DEFL genes in plants infected by *Fusarium* species points to the existence of a conserved common mechanism deployed by the *Fusarium* fungi to inhibit defense responses in plants.

We showed that the elicitors from FS-94 induced expression of a whole panel of 24 DEFL genes in *T. kiharae* seedlings belonging to different structural subgroups (Table S7). Previous studies demonstrated up-regulation of defensin genes by pathogens, abiotic stress, wounding, and symbiosis (Lay & Anderson, 2005; Carvalho & Gomes, 2009; Parisi et al., 2018). In a single study, a defensin gene from *M. truncatula* was shown to be induced in roots in response to infection by the mycorrhizal fungus *Glomus versiforme* (Hanks et al., 2005). Up-regulation of DEFL genes in wheat treated with FS-94 metabolites may reflect activation of the defense system by the elicitors. However, in addition to induction of DEFL genes by the elicitor, a certain portion of DEFLs were down-regulated.

In wheat seedlings pretreated with the elicitor and infected with *F. oxysporum* (IR-expressing seedlings), considerable changes in DEFL expression levels were recorded: expression of 50 DEFL genes changed compared to control seedlings. Of these, 26 DEFL genes were up-regulated and 24 DEFL genes were down-regulated. Six DEFLs were specifically up-regulated in IR-expressing seedlings, namely they were not activated either in elicitor-treated or in infected seedlings. Thus, we may suggest that these DEFLs were primed by FS-94 elicitors for expression after pathogen challenge. Priming, defined as physiological state that enables cells to respond to very low levels of a stimulus in a more strong and rapid manner than non-primed cells, is believed to be a critical process in different types of plant immunity (Conrath, 2011).

Accordingly, we discovered that treatment with *F. sambucinum* elicitors, *F. oxysporum* infection, and infection of elicitor-pretreated *T. kiharae* seedlings results in up-regulation of different sets of DEFL genes. At the same time, different sets of DEFL genes are down-regulated. This may indicate that regulation of wheat DEFL gene expression occurs via different signaling pathways (JA/ET and SA-dependent), and cross-talk between these signaling pathways is observed. The work is now in progress to reveal transcriptional changes in SA, JA and ET-responsive genes in IR-expressing wheat seedlings to reveal signaling pathways involved.

## Conclusion

Our pipelines of DEFL mining proved highly efficient for identification of DEFL transcripts in wheat RNA-seq data. As many as 143 DEFL genes were identified in the wheat *T. kiharae* by global RNA sequencing; the vast majority of them represent novel genes. According to the number of cysteine residues and the cysteine motif, wheat DEFLs were classified into 10 groups. Within these DEFL groups, subgroups of similar sequences can be discriminated. Variation among DEFLs within subgroups is due to amino acid substitutions and insertions/deletions of amino acid sequences. Mutations affecting cysteine residues, which change the number of cysteines and the cysteine motif in wheat DEFLs, were also discovered (see for example, group 9 and 10 DEFLs). Structural diversity of wheat DEFLs is supposed to give rise to their functional diversity. Most discovered DEFLs have a role in plant defense. This follows from their sequence similarity with antimicrobial defensins of other cereals and from up-regulation of their genes in response to fungal infection and treatment with IR elicitors. After confirmation of antimicrobial activity of wheat DEFLs by direct assays, future challenge is to identify the role of sequence variation in pathogen specificity. However, we cannot exclude that some identified DEFLs that were non-responsive to the above-mentioned treatments (e.g., group 2 DEFLs) or were down-regulated have non-defensive functions. In addition to structural diversification, transcriptional diversity of DEFL genes in wheat in response to the fungus, FS-94 elicitors, and the combination of both (elicitors + fungus) was demonstrated, with specific sets of DEFL genes up- and down-regulated in response to different treatments. Variation in expression patterns even between genes encoding structurally similar peptides was detected. We may suggest that transcriptional diversification of wheat DEFLs adds to their functional diversity. Thus, we showed that DEFLs in wheat represent a natural library of structurally and eventually functionally diverse biologically active peptides. Transcriptome profiling of wheat allowed us to gain insight into the mode of action of the elicitors from *F. sambucinum* strain FS-94*.* We discovered that the elicitors up-regulate a specific set of DEFL genes, which alone or together with other defense molecules including other AMPs contribute to enhanced disease resistance of the elicitor-pretreated plants. In addition to direct antimicrobial action of individual DEFLs, synergistic enhancement of their potency, as shown for AMPs, should also be taken into account. Besides providing a better understanding of the mode of action of the elicitors from FS-94 in controlling diseases, up-regulated IR-specific DEFL genes alone or in combination might be used for genetic transformation of plants to develop disease-resistant crops.

##  Supplemental Information

10.7717/peerj.6125/supp-1 Table S1 Primers used for DEFL gene amplification

10.7717/peerj.6125/supp-2 Table S2 Summary of transcriptome assemblies *Combined assembly results from *de novo* assembly of reads from all 4 libraries. For details of clusterization and filtering, see ‘Materials and Methods’.

10.7717/peerj.6125/supp-3 Table S3 Quality evaluation of transcriptome assemblies with BUSCO* *Simão et al. (2015).

10.7717/peerj.6125/supp-4 Table S4 Groups of *T. kiharae* DEFLs based on the number of cysteine residues and cysteine motifs DEFLs are designated as follows: the first figure indicates group number, the second, the number of group member. For example, DEFL1-1 means that this DEFL belongs to group 1, and it is the first group member. DEFLs with identical mature defensin domains are colored similarly. Signal peptides predicted by SignalP and by homology with cereal defensins for group 1 DEFLs are shown in low-case letters. Cysteine motifs characteristic to DEFL groups are presented before the sequences belonging to each group. Prediction of antimicrobial properties was performed with CS-AMPPred (Porto, Pires & Franco, 2012). Isoelectric point (PI) for each putative mature defensin was calculated using IPC tool (Kozlowski, 2016). Accession numbers, annotation and identity score for the top BLAST hits in the NCBI database are presented.*DEFL of *F. oxisporum*.

10.7717/peerj.6125/supp-5 Table S5 Domain identification in DEFLs with InterProScan program Only DEFLs with hits in InterProScan are shown.

10.7717/peerj.6125/supp-6 Table S6 Expression patterns of *T. kiharae* DEFL genes DEFLs with identical mature defensin domain are colored similarly. Up-regulated genes are shown in orange, down-regulated genes, in blue. For up-regulated genes, expression fold change was ≥2, for down-regulated genes, ≤0.5 when gene expression values were ≥ minimal expression threshold. Expression values for individual coding sequences were calculated as Counts per Million Mapped Reads (CPM) only for defensin sequences. Minimal expression threshold was defined as the minimal value of maximal CPM value of predicted defensins in four libraries. Abbreviations used: Ind, elicitor treated; Inf, infected with *F. oxysporum*; IR, elicitor-treated and infected with *F. oxysporum* IR-displaying.

10.7717/peerj.6125/supp-7 Table S7 DEFL genes responsive to *F. sambucinum* elicitors, *F. oxysporum* infection and to *F. oxysporum* infection after elicitor treatment compared with control *T. kiharae* seedlings ^(1)^ ^(1)^ Differentially expressed genes are those with an expression fold change ≥2 (up-regulation) or ≤0.5 (down-regulation); ∗*DEFL* genes responsive to *F. sambucinum* elicitors (Ind) compared with control seedlings (Cont); ^∗∗^DEFL genes responsive to *F. oxysporum* infection (Inf) compared with control seedlings (Cont); ^∗∗∗^DEFL genes responsive to *F. oxysporum* infection after elicitor treatment (in IR-expressing seedlings) compared with control seedlings (Cont). DEFL genes up-regulated in all 3 variants are highlighted yellow, DEFL genes down-regulated in all 3 variants are highlighted green. DEFL genes up-regulated only in IR-expressing seedlings (primed by the elicitors) are highlighted blue.

10.7717/peerj.6125/supp-8 Table S8 Up- and down-regulated DEFL genes in IR-expressing *T. kiharae* seedlings compared with *F. sambucinum*-treated and *F. oxysporum*-infected seedlings ^(1)^ (1) Up-regulated DEFL genes are those with an expression fold change ≥2, down-regulated DEFL genes are those with an expression fold change and ≤0.5. ∗Differentially expressed DEFL genes in IR-expressing compared with elicitor-treated seedlings; ^∗∗^Differentially expressed DEFL genes in IR-expressing compared with *F. oxysporum*-infected seedlings. DEFL genes up-regulated in both variants are highlighted yellow. DEFL genes down-regulated in both variants are highlighted green. DEFL genes up-regulated by the elicitors or infection, but down-regulated in IR-expressing seedlings, respectively, are highlighted blue (for elicitor-induced and *F. oxysporum*)-induced up-regulation of DEFL genes, see Table S7.

10.7717/peerj.6125/supp-9 Figure S1 *T. kiharae* seedlings infected with *F. oxysporum* 12 days after inoculation with *F. oxysporum*. (A) Seeds were pretreated with *F. sambucinum* elicitors. (B) Seeds were pretreated with water (control). For details of treatments, see ‘Materials and Methods’. Photo by Larisa A. Shcherbakova.

10.7717/peerj.6125/supp-10 Figure S2 Neighbor-joining phylogenetic tree of group1 *T. kiharae* DEFLs constructed using Vector NTI Advance 9 DEFL main clusters are designated A–F. Figures in parentheses show genetic distances.

10.7717/peerj.6125/supp-11 Figure S3 Neighbor-joining phylogenetic tree of group 1 DEFL-derived *T. kiharae* defensins and selected defensins from other cereals DEFLs with identical mature peptides are marked with ∗. DEFLs with antimicrobial activity are indicated by ∗∗. Plant species names are abbreviated as follows: Tk, * Triticum kiharae;* Zm,* Zea mays;* Sb, *Sb bicolor;* Hv, *Hordeum vulgare subsp. vulgare;* Rs,* Raphanus sativus;* Ta,* Triticum aestivum;* Aet,* Ae. tauschii subsp. tauschii;* Av,* Avena sativa;* Si,* Setaria italica;* La, *Leymus arenarius*; Tt,* Triticum turgidum subsp. durum*; Ec,* Echinochloa crus-galli*; Os,* Oryza sativa Japonica Group*; Ob,* Oryza brachyantha;* Bd,* Brachypodium distachyon;* Tu,* Triticum urartu.* Calculated genetic distances are shown in parentheses after DEFL name.

10.7717/peerj.6125/supp-12 Figure S4 Multiple sequence alignment of mature *T. kiharae* DEFL-derived peptides with selected defensins from other cereals The conserved cysteine residues are shaded black, while identical amino acids are shaded gray. DEFLs with identical mature peptides are marked with *. DEFLs with antimicrobial activity are marked with **. Group 1 DEFLs homologues with high identity score (%) to defensins from other cereal species, as well as subgroups of closely related peptides named A–F are indicated to the right of the alignment. The species are designated as follows: Tk,* Triticum kiharae;* Zm,* Zea mays;* Sb, *Sb bicolor;* Hv, *Hordeum vulgare subsp. vulgare;* Rs,* Raphanus sativus;* Ta,* Triticum aestivum;* Aet,* Ae. tauschii subsp. tauschii;* Av,* Avena sativa;* Si,* Setaria italica;* La, *Leymus arenarius*; Tt,* Triticum turgidum subsp. durum*; Ec,* Echinochloa crus-galli*; Os,* Oryza sativa Japonica Group*; Ob,* Oryza brachyantha;* Bd,* Brachypodium distachyon;* Tu,* Triticum urartu.*

10.7717/peerj.6125/supp-13 Figure S5 Neighbor-joining phylogenetic tree of group 4 *T. kiharae* DEFLs constructed using Vector NTI Advance 9 Calculated genetic distances are given in parentheses.

10.7717/peerj.6125/supp-14 Figure S6 Neighbor-joining phylogenetic tree of *T. kiharae* DEFLs of groups 5–10 constructed using Vector NTI Advance 9 Calculated genetic distances are shown in parentheses.

10.7717/peerj.6125/supp-15 Figure S7 Differentially expressed DEFL genes in *T. kiharae* transcriptomes* *In % to the total number of expressed DEFL genes. Up-regulated genes (expression fold change ≥2) are colored orange; down-regulated DEFL genes (expression fold change ≤0,5) are colored blue; DEFL genes whose expression level did not change are shown in pink. The designations above the figure are as follows: (A) Ind/Cont, elicitor-treated versus control; (B) Inf/Cont, infected versus control, (C) IR/Cont, IR-expressing versus control; (D) IR/Ind, IR-expressing versus elicitor-treated; (E) IR/Inf, IR-expressing versus infected; (F) Inf/Ind, infected versus elicitor-treated.

10.7717/peerj.6125/supp-16 Figure S8 Venn diagram showing the number of *T. kiharae* DEFL genes up- or down- regulated in IR-displaying seedlings compared with elicitor-treated (Ind) and *F. oxysporum*-infected (Inf) seedlings (A) Up-regulated DEFL genes. (B) Down-regulated DEFL genes. For up-regulated DEFL genes, expression fold change was ≥2, for down-regulated, ≤0.5.

## Additional Information and Declarations

### DNA Deposition

The following information was supplied regarding the deposition of DNA sequences: The Triticum kiharae DEFL sequences are presented in Table S4.

### Data Availability

The following information was supplied regarding data availability: GenBank: SR7511483–SRR7511486.
