# Supplementary material for: Defensin-like peptides in wheat analyzed by whole-transcriptome sequencing: a focus on structural diversity and role in induced resistance
Source: PeerJ. 2019 Jan 8;7:e6125. doi: 10.7717/peerj.6125 (PMC6329339; doi:10.7717/peerj.6125)
Supplement: Table S2 — *Combined assembly results from de novo assembly of reads from all 4 libraries. For details of clusterization and filtering, see ‘Materials and Methods’. [file peerj-07-6125-s002.docx]

**Table S2.** Summary of transcriptome assemblies.

| Assembly | Number of contigs | N50 (b.p.) | Mean contig length (b.p.) | Median contig length (b.p.) |
| --- | --- | --- | --- | --- |
| Control | 133177 | 1133 | 748 | 479 |
| Infected | 125595 | 1214 | 783 | 499 |
| Induced | 156808 | 1208 | 781 | 494 |
| IR-displaying | 149279 | 1241 | 795 | 504 |
| Combined* | 93067 | 1679 | 1333 | 1119 |
| Clusterized all 5 assemblies | 295768 | 1348 | 864 | 570 |
| Filtered by similarity with *T.aestivum* | 168021 | 1589 | 1148 | 926 |
| Final | 127707 | 1693 | 1351 | 1158 |

*Combined assembly results from *de novo* assembly of reads from all 4 libraries. For details of clusterization and filtering, see Materials and Methods.
