# Supplementary material for: Defensin-like peptides in wheat analyzed by whole-transcriptome sequencing: a focus on structural diversity and role in induced resistance
Source: PeerJ. 2019 Jan 8;7:e6125. doi: 10.7717/peerj.6125 (PMC6329339; doi:10.7717/peerj.6125)
Supplement: Table S3 — *Simão et al. (2015). [file peerj-07-6125-s003.docx]

**Table S3.** Quality evaluation of transcriptome assemblies with BUSCO*.

| Assembly | Complete BUSCOs | Complete and single-copy BUSCOs | Complete and duplicated BUSCOs | Fragmented BUSCOs | Missing BUSCOs |
| --- | --- | --- | --- | --- | --- |
| Control | 1164 | 649 | 515 | 124 | 152 |
| Infected | 1150 | 688 | 462 | 123 | 167 |
| Induced | 1190 | 635 | 555 | 118 | 132 |
| Infected and induced | 1195 | 642 | 553 | 112 | 133 |
| Combined | 1262 | 528 | 734 | 85 | 93 |

*****Simão et al., 2015
