# Supplementary material for: Defensin-like peptides in wheat analyzed by whole-transcriptome sequencing: a focus on structural diversity and role in induced resistance
Source: PeerJ. 2019 Jan 8;7:e6125. doi: 10.7717/peerj.6125 (PMC6329339; doi:10.7717/peerj.6125)
Supplement: Table S7 — (1) Differentially expressed genes are those with an expression fold change ≥2 (up-regulation) or ≤0.5 (down-regulation); ∗DEFL genes responsive to F. sambucinum elicitors (Ind) compared with control seedlings (Cont); ∗∗DEFL genes responsive to F. oxysporum infection (Inf) compared with control seedlings (Cont); ∗∗∗DEFL genes responsive to F. oxysporum infection after elicitor treatment (in IR-expressing seedlings) compared with control seedlings (Cont). DEFL genes up-regulated in all 3 variants are highlighted yellow, DEFL genes down-regulated in all 3 variants are highlighted green. DEFL genes up-regulated only in IR-expressing seedlings (primed by the elicitors) are highlighted blue. [file peerj-07-6125-s007.docx]

**Supplemental Table S7.** DEFL genes responsive to *F. sambucinum* elicitors, *F. oxysporum* infection and to *F. oxysporum* infection after elicitor treatment compared with control *T. kiharae* seedlings^(1)^.

| Ind/Cont* | | Inf/Cont** | | IR/Cont*** | |
| --- | --- | --- | --- | --- | --- |
| Up-regulated | Down-regulated | Up-regulated | Down-regulated | Up-regulated | Down-regulated |
| 1-1 | 1-3 | 1-11 | 1-3 | 1-1 | 1-4 |
| 1-14 | 1-4 | 1-12 | 1-4 | 1-2 | 1-10 |
| 1-16 | 1-10 | 1-32 | 1-8 | 1-11 | 1-22 |
| 1-29 | 1-37 | 1-43 | 1-10 | 1-12 | 1-37 |
| 1-30 | 4-2 | 4-6 | 1-22 | 1-16 | 1-44 |
| 1-34 | 4-4 | 4-7 | 1-37 | 1-25 | 4-2 |
| 1-36 | 4-12 | 4-8 | 1-44 | 1-28 | 4-3 |
| 1-39 | 4-18 | 4-28 | 3-4 | 1-32 | 4-5 |
| 1-50 | 4-19 | 7-3 | 4-36 | 1-34 | 4-7 |
| 3-1 | 4-33 |  | 4-40 | 1-36 | 4-10 |
| 3-2 | 4-36 |  | 4-42 | 1-39 | 4-12 |
| 4-7 | 4-37 |  | 5-7 | 1-41 | 4-13 |
| 4-8 | 4-38 |  | 7-2 | 1-43 | 4-18 |
| 4-11 | 4-40 |  | 8-1 | 1-50 | 4-19 |
| 4-17 | 4-42 |  |  | 3-1 | 4-27 |
| 4-20 | 5-3 |  |  | 3-2 | 4-33 |
| 4-28 | 7-2 |  |  | 3-5 | 4-36 |
| 4-32 | 8-1 |  |  | 4-7 | 4-37 |
| 4-34 |  |  |  | 4-8 | 4-38 |
| 4-35 |  |  |  | 4-9 | 4-40 |
| 8-2 |  |  |  | 4-20 | 4-41 |
| 10-2 |  |  |  | 4-28 | 4-42 |
| 10-4 |  |  |  | 4-32 | 7-2 |
| 10-5 |  |  |  | 4-34 | 8-1 |
|  |  |  |  | 4-35 | 9-2 |
|  |  |  |  | 8-2 |  |

^(1)^ Differentially expressed genes are those with an expression fold change ≥2 (up-regulation) or ≤0.5 (down-regulation); *DEFL genes responsive to *F. sambucinum* elicitors (Ind) compared with control seedlings (Cont); **DEFL genes responsive to *F. oxysporum* infection (Inf) compared with control seedlings (Cont); ***DEFL genes responsive to *F. oxysporum* infection after elicitor treatment (in IR-expressing seedlings) compared with control seedlings (Cont). DEFL genes up-regulated in all 3 variants are highlighted yellow, DEFL genes down-regulated in all 3 variants are highlighted green. DEFL genes up-regulated only in IR-expressing seedlings (primed by the elicitors) are highlighted blue.
