# Supplementary material for: Defensin-like peptides in wheat analyzed by whole-transcriptome sequencing: a focus on structural diversity and role in induced resistance
Source: PeerJ. 2019 Jan 8;7:e6125. doi: 10.7717/peerj.6125 (PMC6329339; doi:10.7717/peerj.6125)
Supplement: Table S8 — (1) Up-regulated DEFL genes are those with an expression fold change ≥2, down-regulated DEFL genes are those with an expression fold change and ≤0.5. ∗Differentially expressed DEFL genes in IR-expressing compared with elicitor-treated seedlings; ∗∗Differentially expressed DEFL genes in IR-expressing compared with F. oxysporum-infected seedlings. DEFL genes up-regulated in both variants are highlighted yellow. DEFL genes down-regulated in both variants are highlighted green. DEFL genes up-regulated by the elicitors or infection, but down-regulated in IR-expressing seedlings, respectively, are highlighted blue (for elicitor-induced and F. oxysporum)-induced up-regulation of DEFL genes, see Table S7. [file peerj-07-6125-s008.docx]

**Supplemental Table S8.** Up- and down-regulated DEFL genes in IR-expressing *T. kiharae* seedlings compared with *F. sambucinum*-treated and *F. oxysporum*-infected seedlings^(1)^.

| IR/Ind* | | IR/Inf** | |
| --- | --- | --- | --- |
| Up-regulated DEFL genes | Down-regulated DEFL genes | Up-regulated regulated DEFL genes | Down-regulated regulated DEFL genes |
| DEFL1-2 | DEFL1-6 | DEFL1-1 | DEFL1-24 |
| 1-11 | 1-7 | 1-3 | 4-2 |
| 1-12 | 1-9 | 1-5 | 4-3 |
| 1-25 | 1-14 | 1-12 | 4-5 |
| 1-27 | 1-22 | 1-20 | 4-6 |
| 1-28 | 1-29 | 1-23 | 4-10 |
| 1-32 | 1-30 | 1-34 | 4-12 |
| 1-41 | 1-33 | 1-39 | 4-13 |
| 1-43 | 1-35 | 1-40 | 4-18 |
| 4-4 | 1-44 | 1-41 | 4-19 |
| 4-15 | 1-52 | 1-43 | 4-27 |
|  | 4-3 | 1-45 | 4-33 |
|  | 4-5 | 1-48 | 4-37 |
|  | 4-7 | 1-50 | 4-38 |
|  | 4-11 | 3-1 | 4-41 |
|  | 4-13 | 3-4 | 7-3 |
|  | 4-19 | 3-5 |  |
|  | 4-27 | 4-20 |  |
|  | 4-38 | 4-35 |  |
|  | 9-2 | 5-1 |  |
|  | 10-1 | 5-12 |  |
|  | 10-2 | 6-2 |  |
|  | 10-3 |  |  |
|  | 10-4 |  |  |
|  | 10-5 |  |  |
|  | 10-6 |  |  |

^(1)^ Up-regulated DEFL genes are those with an expression fold change ≥2, down-regulated DEFL genes are those with an expression fold change and ≤0.5. *Differentially expressed DEFL genes in IR-expressing compared with elicitor-treated seedlings; **Differentially expressed DEFL genes in IR-expressing compared with *F. oxysporum*-infected seedlings. DEFL genes up-regulated in both variants are highlighted yellow. DEFL genes down-regulated in both variants are highlighted green. DEFL genes up-regulated by the elicitors or infection, but down-regulated in IR-expressing seedlings, respectively, are highlighted blue (for elicitor-induced and *F. oxysporum*-induced up-regulation of DEFL genes, see Table S7).
