# Supplementary figures and images for: Defensin-like peptides in wheat analyzed by whole-transcriptome sequencing: a focus on structural diversity and role in induced resistance
Source: PeerJ. 2019 Jan 8;7:e6125. doi: 10.7717/peerj.6125 (PMC6329339; doi:10.7717/peerj.6125)

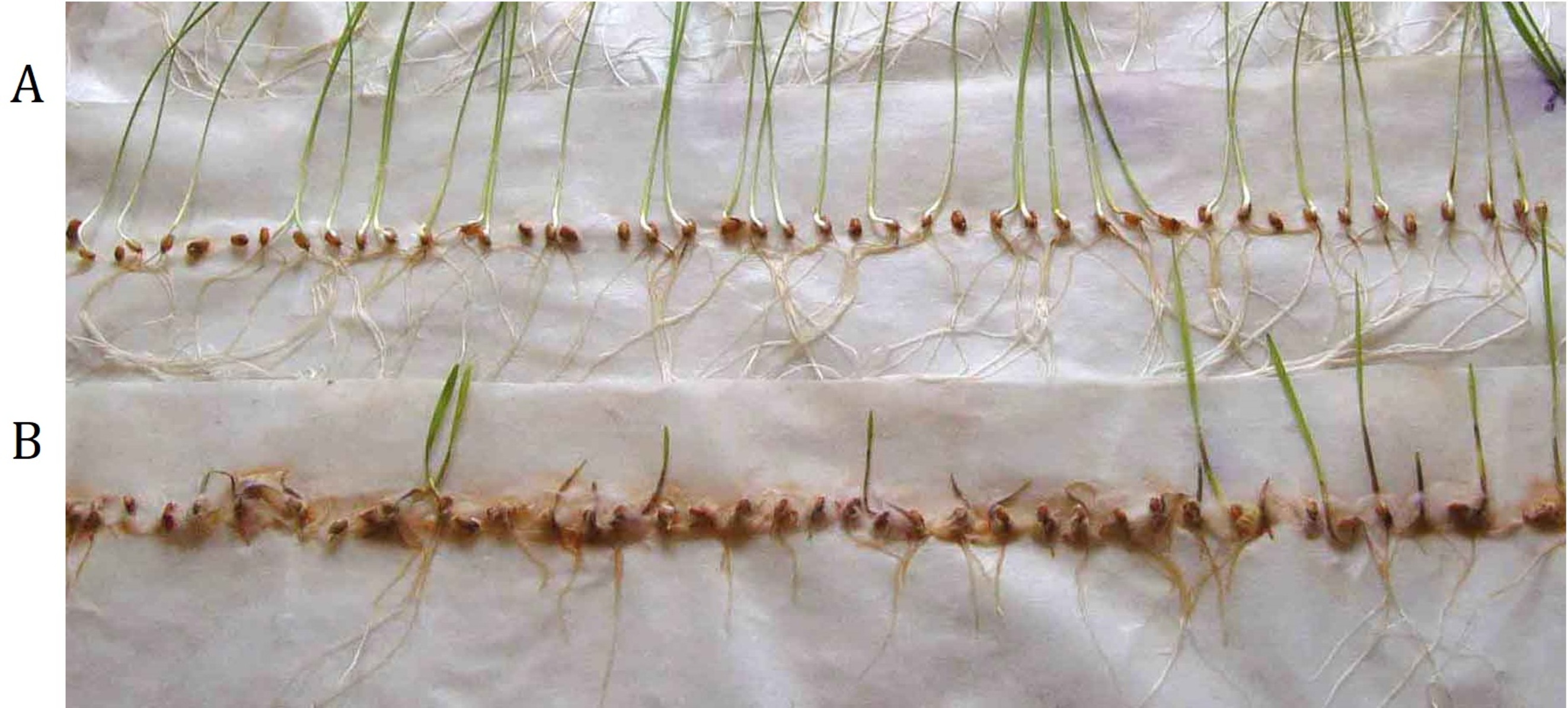

Supplement: Figure S1 — 12 days after inoculation with F. oxysporum. (A) Seeds were pretreated with F. sambucinum elicitors. (B) Seeds were pretreated with water (control). For details of treatments, see ‘Materials and Methods’. Photo by Larisa A. Shcherbakova. [file peerj-07-6125-s009.jpg]

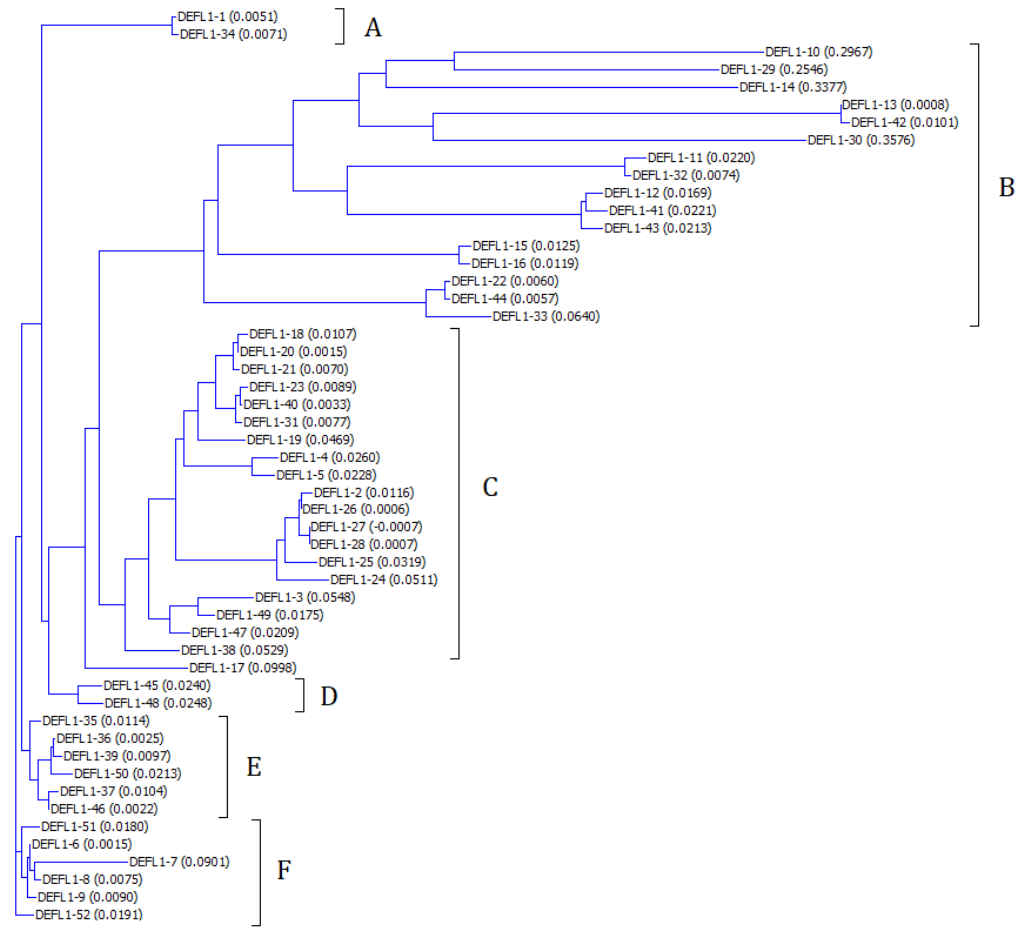

Supplement: Figure S2 — DEFL main clusters are designated A–F. Figures in parentheses show genetic distances. [file peerj-07-6125-s010.png]

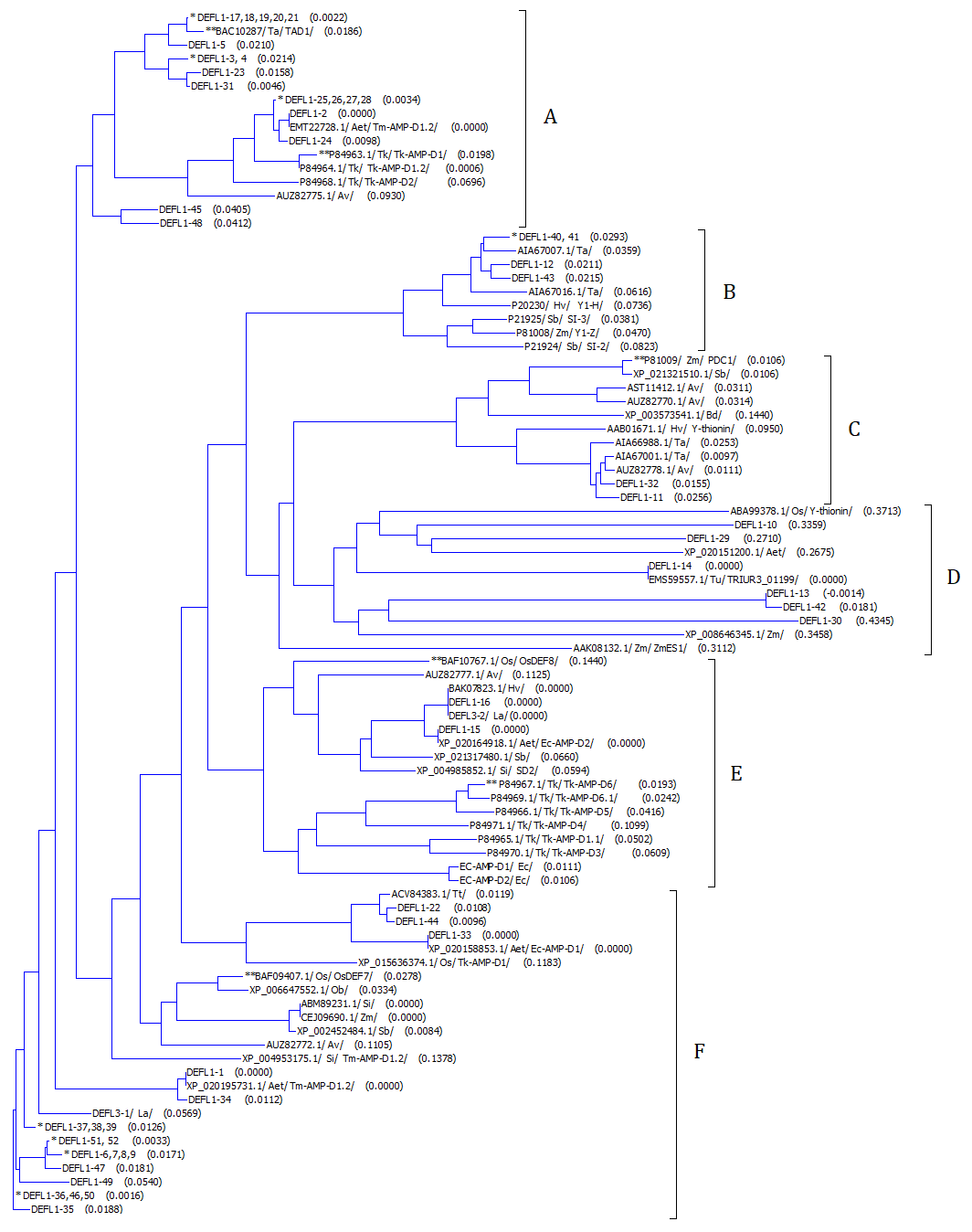

Supplement: Figure S3 — DEFLs with identical mature peptides are marked with ∗. DEFLs with antimicrobial activity are indicated by ∗∗. Plant species names are abbreviated as follows: Tk, Triticum kiharae; Zm, Zea mays; Sb, Sb bicolor; Hv, Hordeum vulgare subsp. vulgare; Rs, Raphanus sativus; Ta, Triticum aestivum; Aet, Ae. tauschii subsp. tauschii; Av, Avena sativa; Si, Setaria italica; La, Leymus arenarius; Tt, Triticum turgidum subsp. durum; Ec, Echinochloa crus-galli; Os, Oryza sativa Japonica Group; Ob, Oryza brachyantha; Bd, Brachypodium distachyon; Tu, Triticum urartu. Calculated genetic distances are shown in parentheses after DEFL name. [file peerj-07-6125-s011.png]

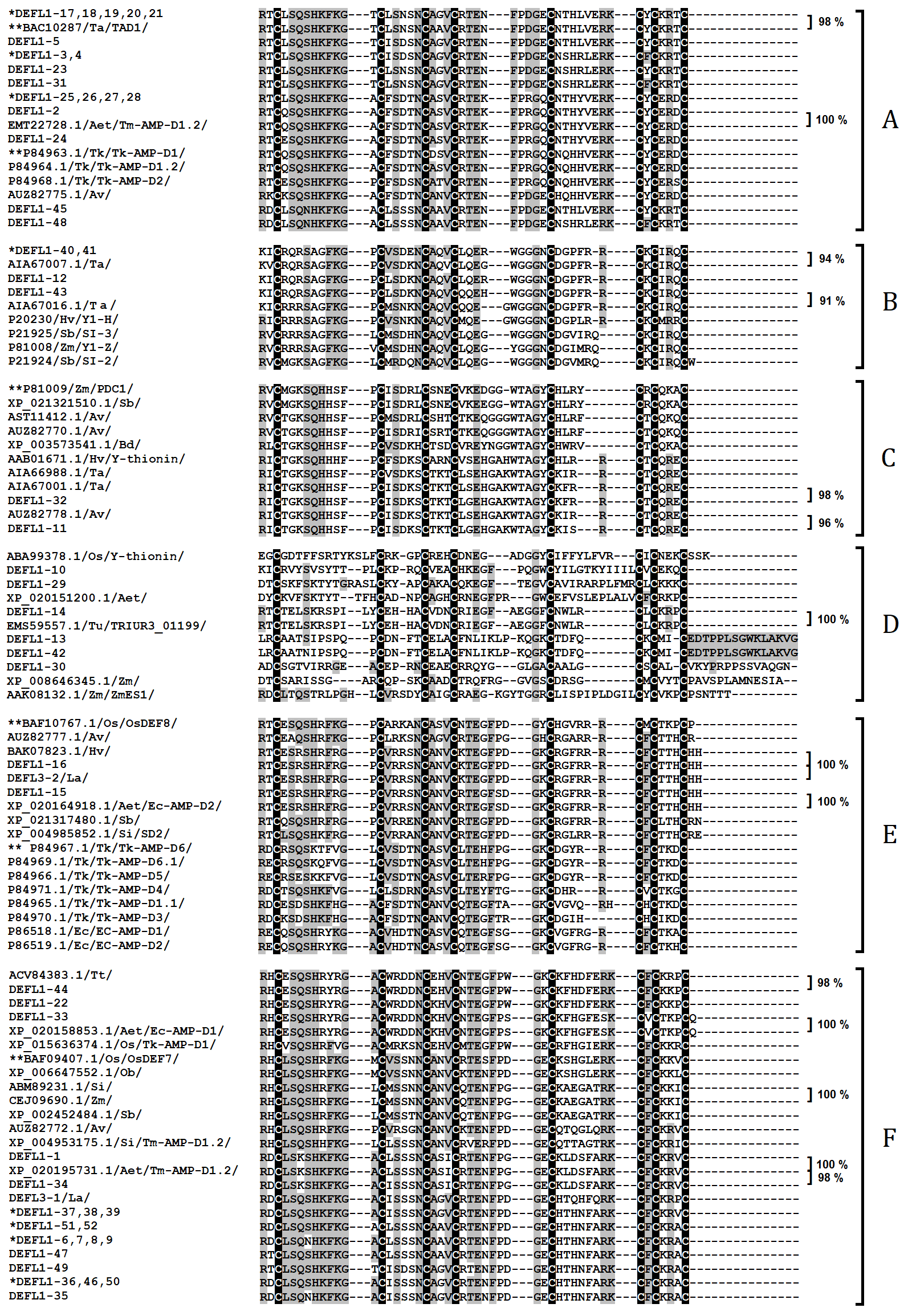

Supplement: Figure S4 — The conserved cysteine residues are shaded black, while identical amino acids are shaded gray. DEFLs with identical mature peptides are marked with *. DEFLs with antimicrobial activity are marked with **. Group 1 DEFLs homologues with high identity score (%) to defensins from other cereal species, as well as subgroups of closely related peptides named A–F are indicated to the right of the alignment. The species are designated as follows: Tk, Triticum kiharae; Zm, Zea mays; Sb, Sb bicolor; Hv, Hordeum vulgare subsp. vulgare; Rs, Raphanus sativus; Ta, Triticum aestivum; Aet, Ae. tauschii subsp. tauschii; Av, Avena sativa; Si, Setaria italica; La, Leymus arenarius; Tt, Triticum turgidum subsp. durum; Ec, Echinochloa crus-galli; Os, Oryza sativa Japonica Group; Ob, Oryza brachyantha; Bd, Brachypodium distachyon; Tu, Triticum urartu. [file peerj-07-6125-s012.png]

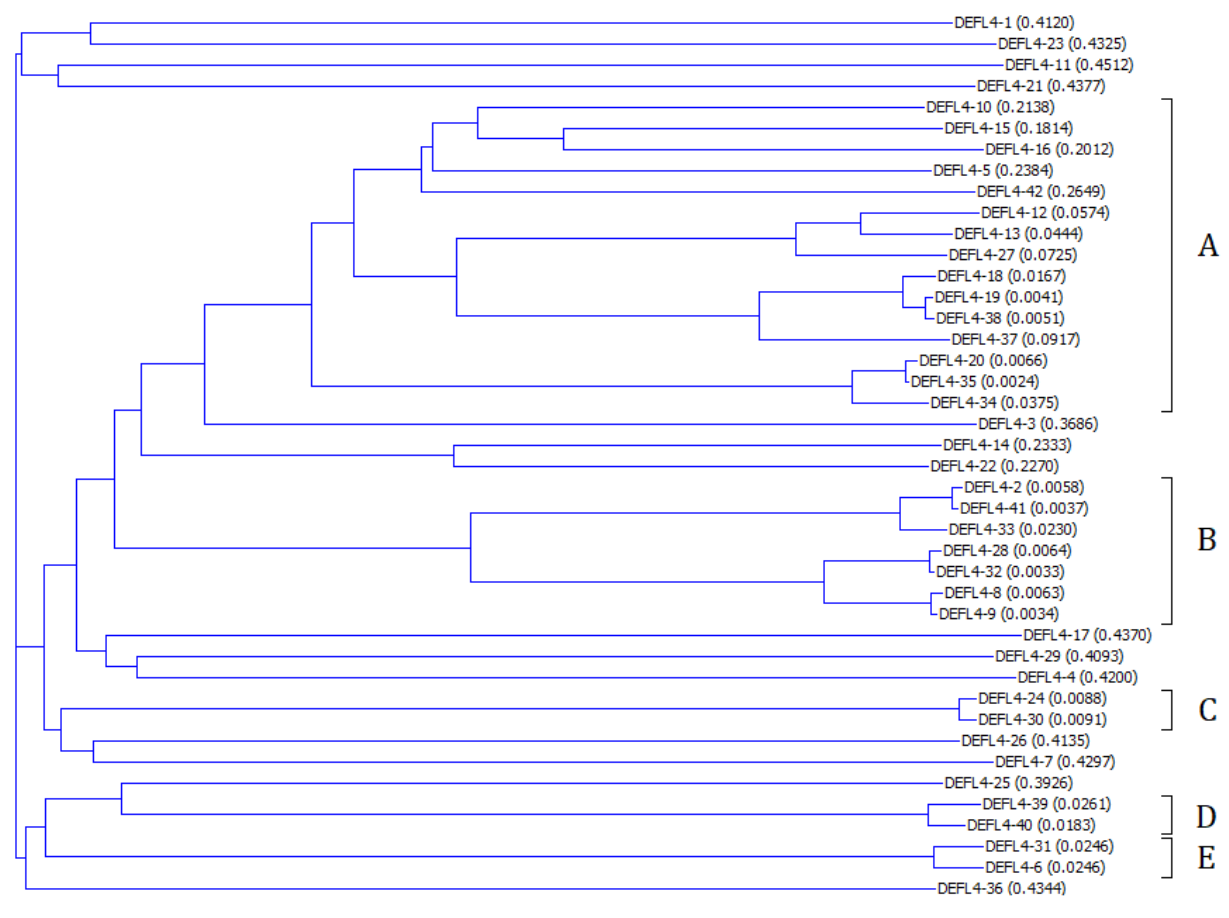

Supplement: Figure S5 — Calculated genetic distances are given in parentheses. [file peerj-07-6125-s013.png]

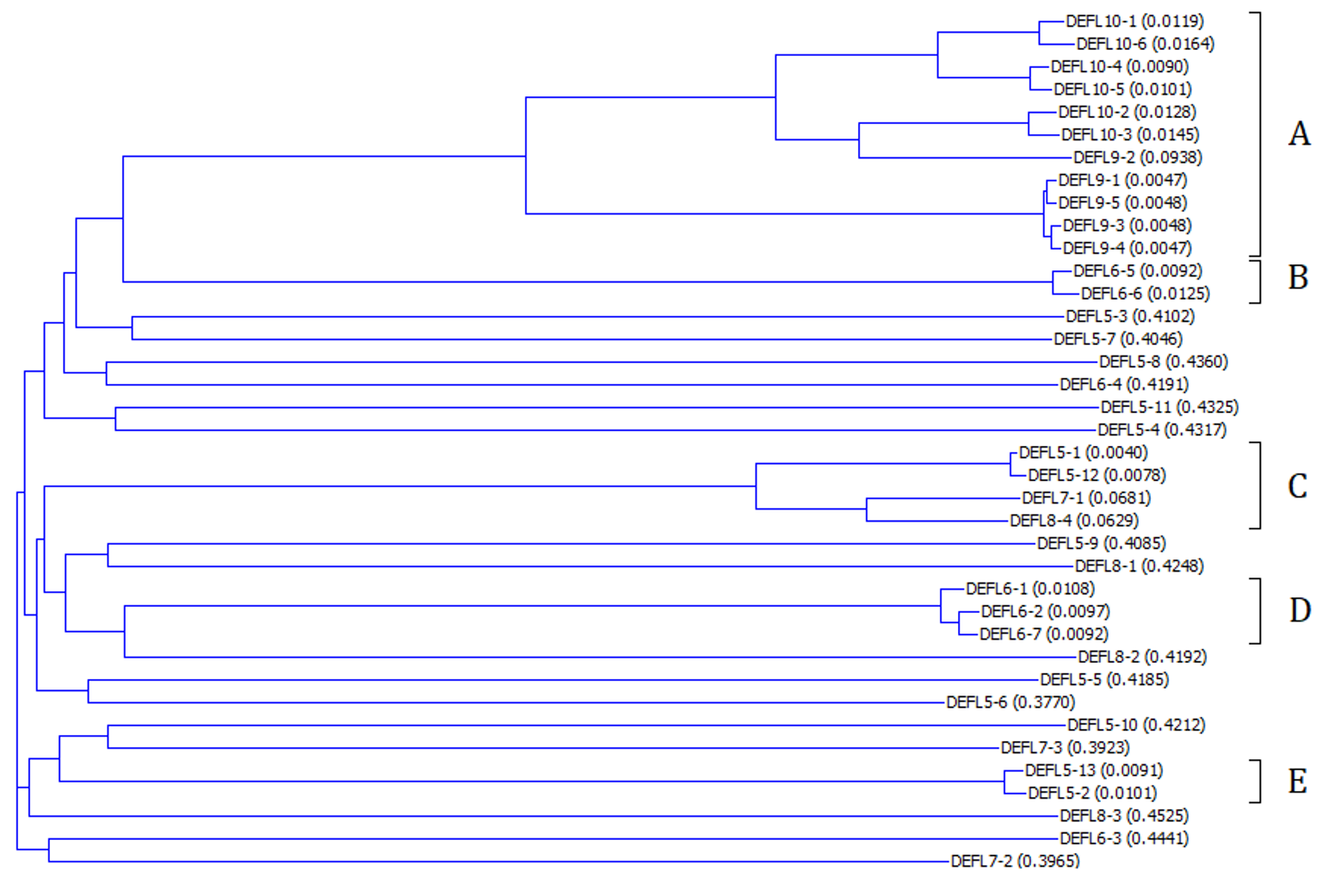

Supplement: Figure S6 — Calculated genetic distances are shown in parentheses. [file peerj-07-6125-s014.png]

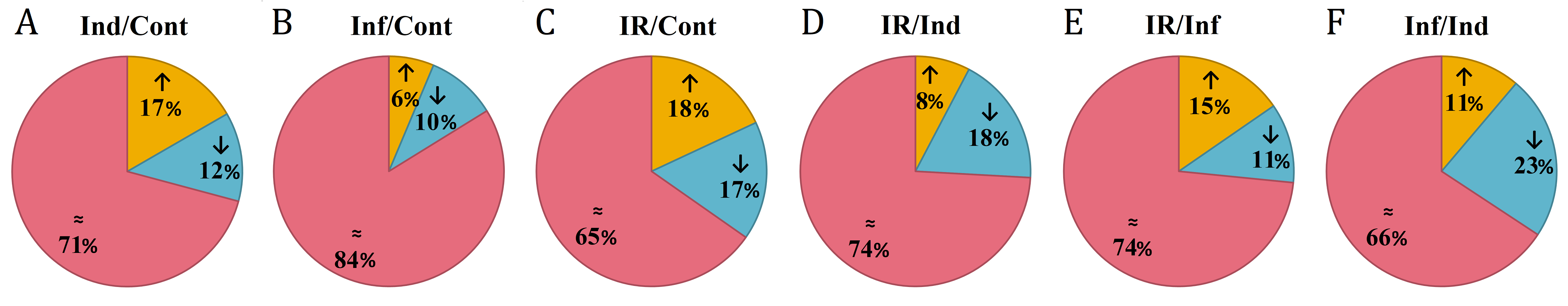

Supplement: Figure S7 — *In % to the total number of expressed DEFL genes. Up-regulated genes (expression fold change ≥2) are colored orange; down-regulated DEFL genes (expression fold change ≤0,5) are colored blue; DEFL genes whose expression level did not change are shown in pink. The designations above the figure are as follows: (A) Ind/Cont, elicitor-treated versus control; (B) Inf/Cont, infected versus control, (C) IR/Cont, IR-expressing versus control; (D) IR/Ind, IR-expressing versus elicitor-treated; (E) IR/Inf, IR-expressing versus infected; (F) Inf/Ind, infected versus elicitor-treated. [file peerj-07-6125-s015.png]

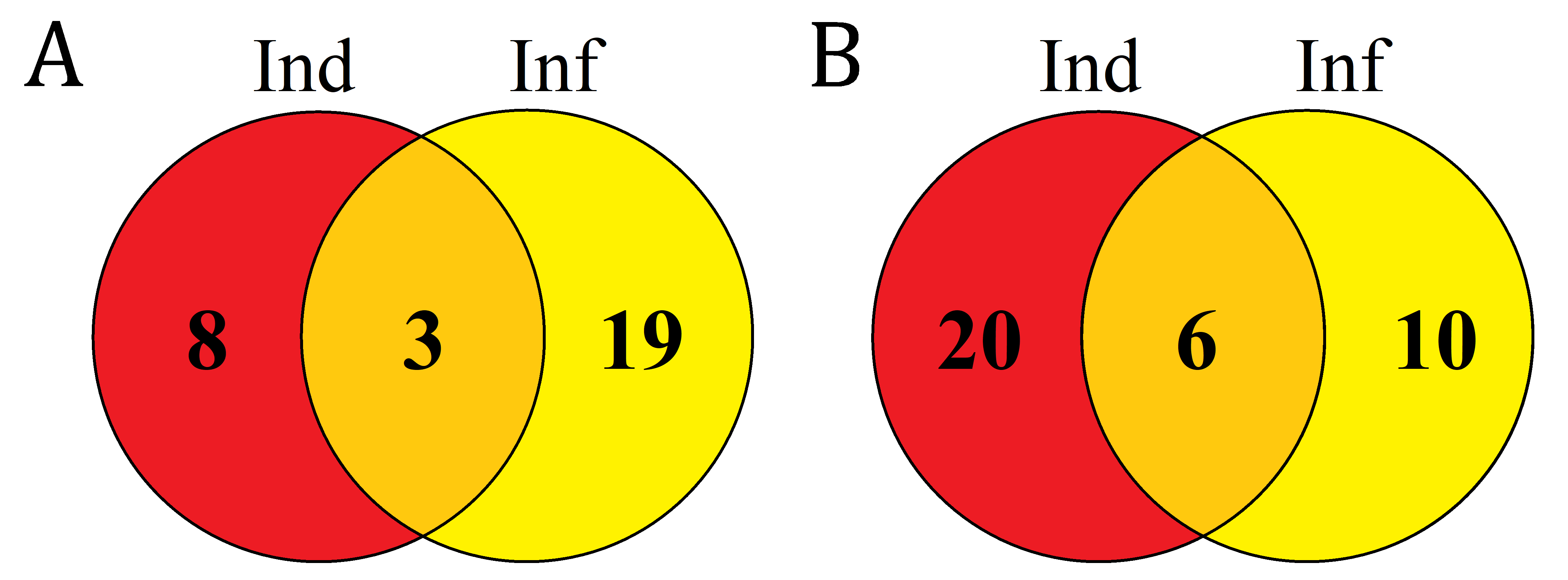

Supplement: Figure S8 — (A) Up-regulated DEFL genes. (B) Down-regulated DEFL genes. For up-regulated DEFL genes, expression fold change was ≥2, for down-regulated, ≤0.5. [file peerj-07-6125-s016.png]
